# Respiratory and systemic impacts following MWCNT inhalation in B6C3F1/N mice

**DOI:** 10.1186/s12989-021-00408-z

**Published:** 2021-03-26

**Authors:** Christopher T. Migliaccio, Raymond F. Hamilton, Pamela K. Shaw, Joseph F. Rhoderick, Sanghamitra Deb, Rohit Bhargava, Jack R. Harkema, Andrij Holian

**Affiliations:** 1 Department of Biomedical and Pharmaceutical Sciences, Center for Environmental Health Sciences, University of Montana, Missoula, MT 59812 USA; 2 Department of Chemistry and Biochemistry, Alabama Analytical Research Center, University of Alabama, Tuscaloosa, AL 35487 USA; 3 Beckman Institute, University of Illinois at Urbana-Champaign, Urbana, IL 61801 USA; 4 Department of Pathobiology and Diagnostic Investigation, Michigan State University, East Lansing, MI 48824 USA

## Abstract

**Background:**

A very pure multi-walled carbon nanotube (MWCNT) that was shown to have very low toxicity in vitro, was evaluated for lung and systemic effects and distribution following inhalation exposure.

**Methods:**

B6C3F1/N mice were exposed to varying doses (0, 0.06, 0.2, and 0.6 mg/m^3^) of the (99.1% carbon) MWCNT by inhalation for 30 days (excluding weekends). Ten days following the last exposure, the lungs and spleen were harvested and processed for histology and immune cell population assessment. In addition, lung lavage cells and fluid were analyzed. Stimulated Raman scattering (SRS) was used to identify particles in the lungs, spleen, kidneys, liver, mediastinal and brachial lymph nodes, and olfactory bulb. Splenic tissue sections were stained with hematoxylin and eosin (H&E) for light microscopic histopathology assessment. Blood plasma was analyzed for cytokines and cathepsins. A section of the spleen was processed for RNA isolation and relative gene expression for 84 inflammation-related cytokines/chemokines.

**Results:**

Following MWCNT exposure, particles were clearly evident in the lungs, spleens, lymph nodes and olfactory bulbs, (but not livers or kidneys) of exposed mice in a dose-dependent manner. Examination of the lavaged lung cells was unremarkable with no significant inflammation indicated at all particle doses. In contrast, histological examination of the spleen indicated the presence of apoptotic bodies within T cells regions of the white pulp area. Isolated splenic leukocytes had significant changes in various cells including an increased number of proinflammatory CD11b^+^Ly6C^+^ splenic cells. The gene expression studies confirmed this observation as several inflammation-related genes were upregulated particularly in the high dose exposure (0.6 mg/m^3^). Blood plasma evaluations showed a systemic down-regulation of inflammatory cytokines and a dose-dependent up-regulation of lysosomal cathepsins.

**Conclusions:**

The findings in the lungs were consistent with our hypothesis that this MWCNT exposure would result in minimal lung inflammation and injury. However, the low toxicity of the MWCNT to lung macrophages may have contributed to enhanced migration of the MWCNT to the spleen through the lymph nodes, resulting in splenic toxicity and systemic changes in inflammatory mediators.

**Supplementary Information:**

The online version contains supplementary material available at 10.1186/s12989-021-00408-z.

## Background

The increasing use of engineered nanomaterials in biomedical and technological applications, necessitates a comprehensive investigation of their impacts on human health. The most common routes of nanomaterial exposures are by inhalation, ingestion and potential absorption through the skin [1]. Of these, most research has involved inhalation exposure and subsequent lung inflammation [2]. One of the most common and extensively investigated nanomaterials are multi-walled carbon nanotubes (MWCNT). However, there are a number of significant challenges in arriving at any generalized conclusions in testing potential health effects of MWCNT. Many of these have been discussed before [3], but a few challenges include but not limited to: 1) selecting from a wide assortment of MWCNT (e.g., length, width, and presence of metal contaminants) in the market; 2) determining what model system to use (in vitro what cells and in vivo what species and strains); 3) choosing what dose(s) to test and what in vivo exposure method to use (instillation or inhalation); 4) MWCNT agglomeration in liquids or air; and 5) selecting what responses/endpoints to evaluate. By selectively choosing materials, models, doses and endpoints, conclusions about the potential health risks from MWCNT exposures could range from benign to severe.

Studies using respiratory exposure (either instillation or inhalation) have shown that key target cells in the lung are most often epithelial cells and macrophages. Since macrophages are the primary innate immune cells responsible for maintaining lung homeostasis and removal of inhaled particles, it is expected that they would take up significant quantities of MWCNT [4]. Furthermore, macrophages are key inflammatory cells in the lung and are capable of releasing significant quantities of various inflammatory cytokines including IL-1β and IL-18 as a result of NLRP3 Inflammasome and caspase-1 activation; early steps in the inflammatory response [5, 6]. Caspase-1 activation may also contribute to macrophage cytotoxicity through pyroptosis [7]. In order to address the challenges previously described, a panel of 24 MWCNT provided by the National Toxicology Program were tested using differentiated THP-1 cells as an in vitro model of macrophage responses [8]. The toxic and inflammatory responses ranged from minimal to significant. Additional studies using murine alveolar macrophages in vitro and in vivo studies confirmed that the MWCNT activity appeared to be correlated with the amount of Ni present on the MWCNT [9]. In addition, pristine MWCNT had minimal impact on lung inflammation.

Because inhalation is the primary route of exposure, many of the in vivo studies with MWCNT have focused on lung inflammation and pathology [10–12]. Although it is known that nanomaterials can redistribute to other tissues following lung exposure [13–15], the mechanisms of redistribution to secondary organs have not been defined. Furthermore, the impacts of these materials on secondary organs and systemic effects have received limited attention. Due to the nature of particle interactions with immune cells (macrophages) following inhalation, it is reasonable to anticipate immune translocation of particles as a mechanism of systemic effects. Of note, the spleen is a common tissue used to assess systemic markers of disease progression (i.e. cancer) or inhalation exposures [16–18].

The current study was part of a larger inhalation exposure protocol conducted by The National Toxicology Program using B6C3F1/N mice. Whole body inhalation exposures were conducted at Battelle Laboratories using B6C3F1/N mice exposed to three aerosol concentrations of Long Multiwalled nanotubes 10 μm long, 20 nm in diameter (L-MWNT-1020) or filtered air for 30 days (excluding weekends). The mice were subsequently distributed to a number of laboratories for investigation. L-MWNT-1020 was selected because of its high purity with minimal metal contamination. The strain of mice was selected based on the interest of The National Toxicology Program to evaluate potential carcinogenic effects [19]. One of the recipient laboratories evaluated impacts on allergy reporting decreased Th2 responses [20]. Based on the purity of the MWCNT and previous work, we hypothesized that this MWCNT would have minimal adverse impacts in vivo in the lung presenting an opportunity to examine what would be expected to be minimal responses in an animal model and establish a baseline for comparison studies with other MWCNT. Since there is little published research concerning the systemic effects of these types of MWCNT, we conducted a detailed evaluation of systemic distribution and systemic impacts.

## Results

### Lung

#### MWCNT in the lung following exposures

**Fig. 1:**
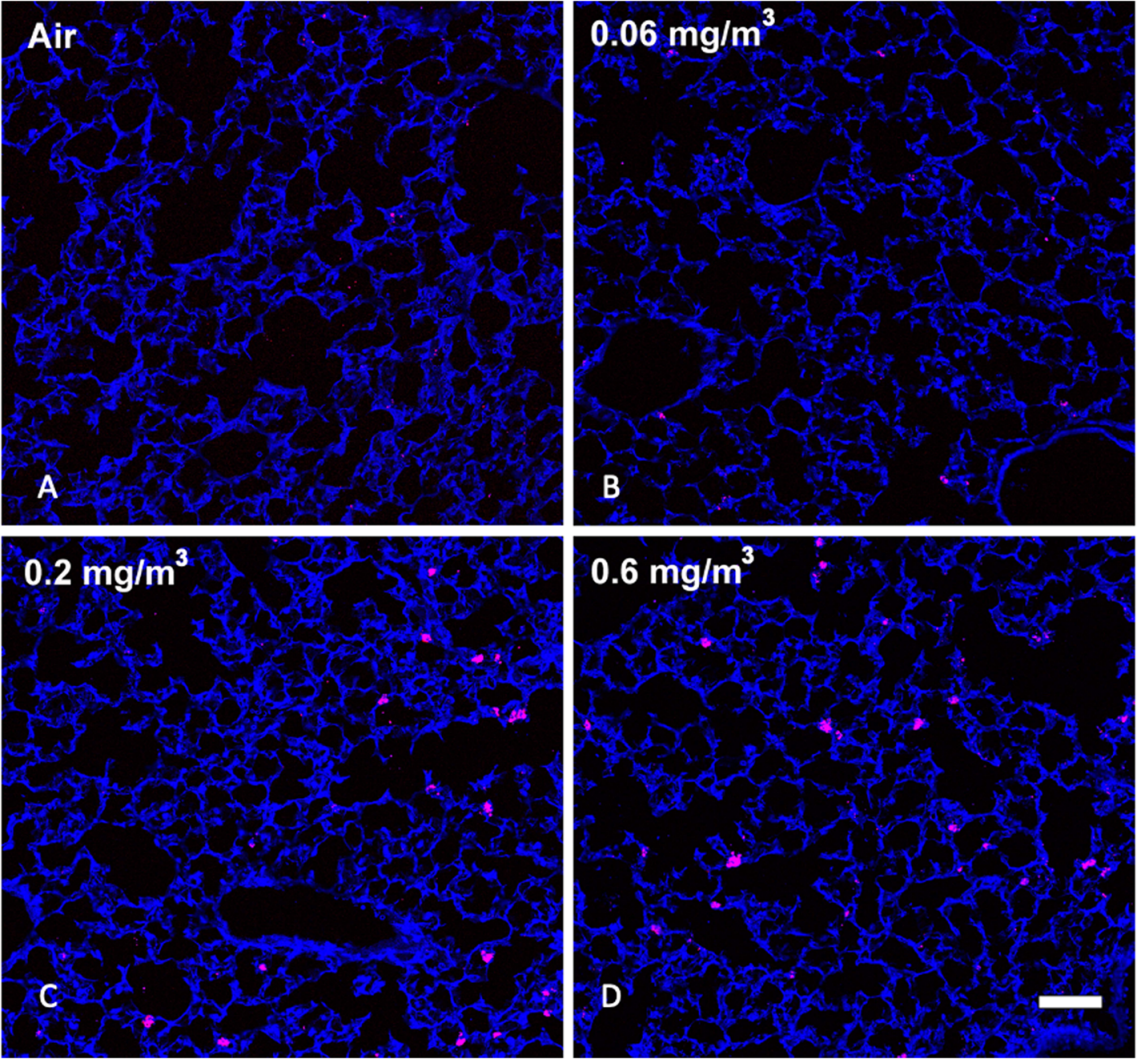
*Representative MWCNT particle deposition in lung tissues following 30-day exposure + 10 days recovery with all exposure groups shown.* Lung tissue was assessed for MWCNT particle deposition using stimulated raman scattering (SRS) spectroscopy, where the pink color represents detected particles. The above images are representative of sampled lung sections from **a** Air control or 0 mg/m^3^, **b** 0.06 mg/m^3^, **c** 0.2 mg/m^3^, and **d** 0.6 mg/m^3^. Blue = normal tissue. Pink = MWCNT particle deposition. White scale bar = 50 μm

Based on previous work and published observations [10], it was expected that prolonged inhalation exposures to MWCNT would lead to detectable particle deposition in the lung that would be retained long-term. Examination of MWCNT in lung tissue was determined using stimulated Raman scattering (SRS) in large sections of lung tissue [10]. Figure 1 shows SRS images of lung sections from control and particle-exposed for each of the conditions: A) Air control or 0 mg/m^3^, B) 0.06 mg/m^3^, C) 0.2 mg/m^3^, and D) 0.6 mg/m^3^ mice following 30 days of exposure (see Methods) plus 10 days post exposure. In general, there was a dose-dependent increase of MWCNT deposition in the lungs, although the increase at the highest dose did not appear to be 3x that of the middle dose. In addition, the MWCNT was relatively uniformly dispersed in the lung tissue. The particle deposition was most obvious at the two highest MWCNT doses, exhibiting small and larger aggregates of MWCNT.

#### Lung macrophages

**Fig. 2:**
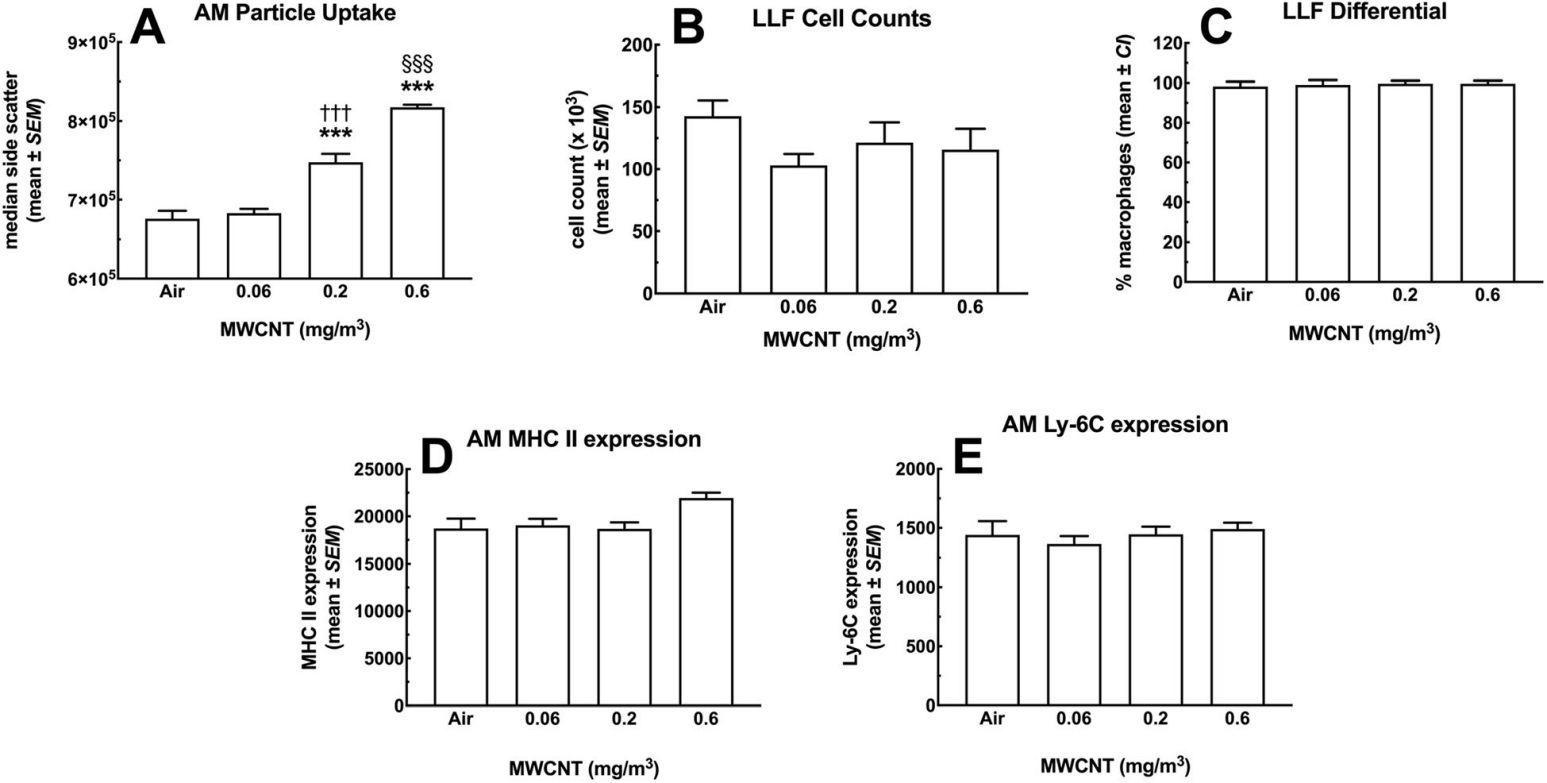
*MWCNT effects on alveolar macrophages following 30-day exposure + 10 days recovery retrieved by lung lavage.* **a** Increase in AM side scatter (SSC) follows the MWCNT dose-response, indicating the particle was still in the lungs 40 days from the start of the exposure and 10 days from the last exposure. *** indicates *P* < 0.001 compared to Air control and low dose. §§§ *P* < 0.001 compared to all other groups. **b** LLF total cell counts **c** AM percent of total cells. **d** AM MHCII expression (relative median fluorescence intensity). **e** AM Ly-6c expression (relative median fluorescence intensity). All data expressed as mean ± *SEM* (*n* = 4 pairs)

Alveolar macrophages (AM) are the primary innate immune cells responsible for phagocytosing inhaled particles. Therefore, AM retrieved from the lung lavage fluid (LLF) of the various dose groups were examined for MWCNT particle content using side scatter (SSC) (Fig. 2a). The increased side scatter was proportional to dose, thus, AM MWCNT content appeared to accurately represent the dose-dependent exposures. SSC indicated significant increases in particles above the lowest inhalation dose. These results were consistent with the Raman results showing a dose-dependent MWCNT in the lung tissue with increasing doses of exposure. Images of isolated AM can be seen in Supplemental Figure S1.

Based on previous research with the MWCNT under investigation, minimal effects on AM toxicity/count were expected (unpublished observations). Figure 2b shows the cell count from lung lavage showing no significant differences regardless of exposure conditions. Figure 2c indicates that the lavage cells were mostly AM determined by differential counting at slightly less than 96% in every experimental group. These findings suggest that either there was no acute inflammation from particle exposure and polymorphonuclear neutrophil (PMN) influx, or any initial response had resolved in the time since the start of the experiment to the collection of LLF.

In order to further classify the AM, specific markers were examined as shown in Figs. 2d – e. Figure 2d shows the major histocompatibility complex II (MHCII) marker, associated with antigen presentation (APC). There was a slight, nonsignificant increase as a result of the highest MWCNT dose, but otherwise no significant changes were noted. Likewise, compared to the air control AM, the differentiation marker Ly-6C did not change on AM retrieved from mice exposed to MWCNT, regardless of dose (Fig. 2e). Taken together, these results suggest no significant alterations to the AM populations in MWCNT-exposed animals consistent with the proposal that these MWCNT did not cause marked lung inflammation with respect to changes in lung cell populations.

#### Lung lavage fluid cytokines

**Fig. 3:**
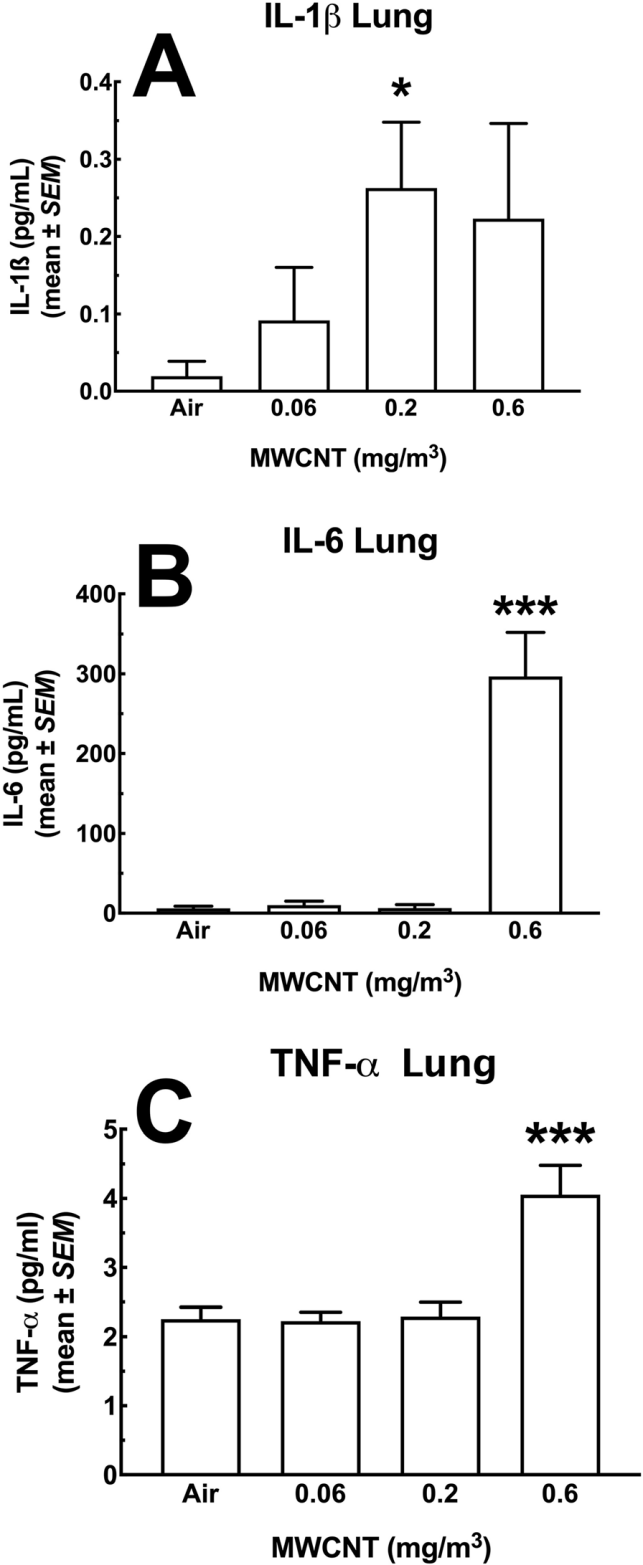
*Cytokine changes in the lung lavage fluid of mice exposed to MWCNT following 30-day exposure + 10 days recovery.* The first pull of cold saline lavage fluid was used to assess a panel of seven analytes. The MesoScale Discovery (MSD) system was used to assay for multiple analytes in a single sample. In the figure above, there was a significant observed increase in three of the seven inflammatory mediators including **a** IL-1β, **b** IL-6, and **c** TNF-α. Asterisks * indicate statistical significance at *P* < 0.05 compared to ‘Air’ control or *** where *P* < 0.001 compared to all other doses by Holm-Sydak *post-hoc* analyses. All data expressed as mean ± *SEM* (*n* = 8)

**Fig. 4:**
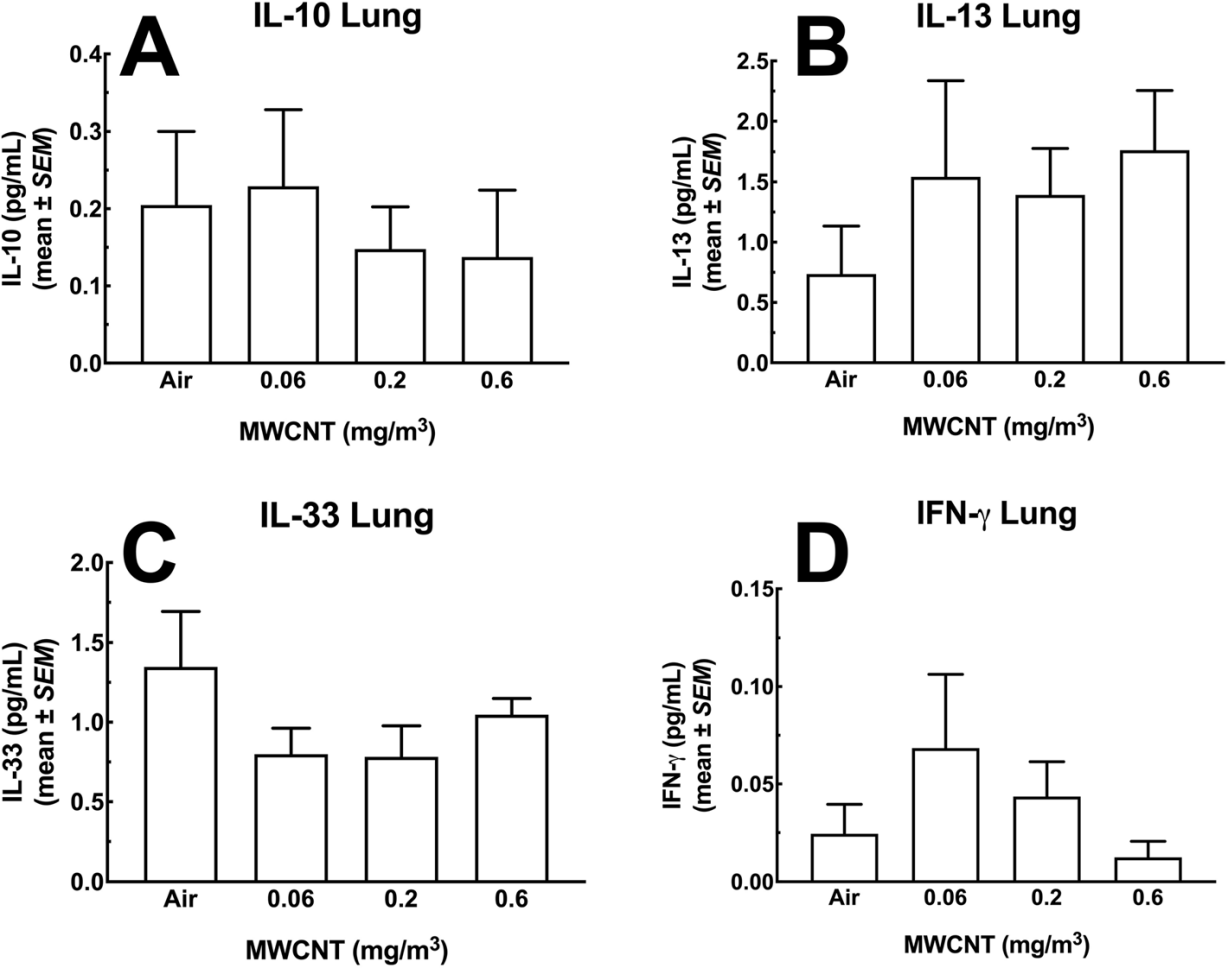
*Cytokines in the lung lavage fluid of mice exposed to MWCNT for 30 days + 10 days recovery.* The first pull of cold saline lavage fluid was used to assess a panel of seven analytes. The MesoScale Discovery (MSD) system was used to assay for multiple analytes in a single sample. In the figure above, there was no significant changes observed in the following cytokines: **a** IL-10, **b** IL-13, **c** IL-33 and **d** IFN-γ. All data expressed as mean ± *SEM* (*n* = 8)

Figure 3a – c show significant increases in a subset of inflammatory cytokines present in the LLF post-MWCNT-exposure. IL-1β levels are shown in Fig. 3a and were substantially higher in samples from the highest 2 doses of particle. The absolute value of IL-1β was very low (sub-picogram/mL), which could be a dilution effect of LLF indicative of a weak response at this time point and/or minimal activation of the NOD-, LRR- and pyrin domain-containing protein 3 (NLRP3) inflammasome [21]. In contrast, IL-6 and TNF-α, seen in Fig. 3b and c respectively, showed a robust response in the highest dose (0.6 mg/m^3^). In contrast to the results shown in Fig. 3a - c, Figs. 4a – d show cytokine levels that were not affected by the MWCNT exposure regardless of dose. IL-10 (4A), IL-13 (4B), IL-33 (4C) and IFN-γ (4D) showed some increases and decreases, but were not statistically significant

#### Draining lymph nodes

*Appearance of MWCNT in the Mediastinal and Brachial Lymph Nodes Following Exposures.*

**Fig. 5:**
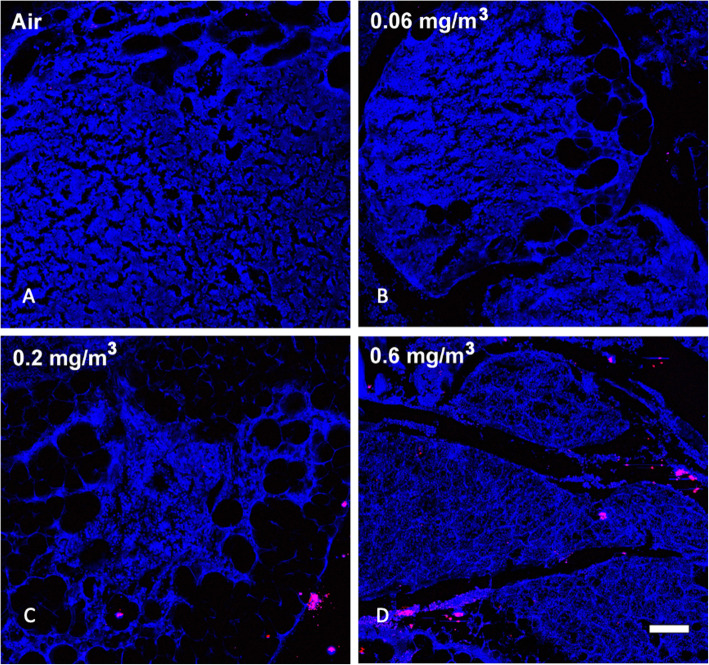
*Representative MWCNT particle deposition in mediastinal lymph node (MLN) tissues following 30-day exposure + 10 days recovery with all exposure groups shown.* MLN tissue was assessed for MWCNT particle deposition using stimulated raman scattering (SRS) spectroscopy, where the pink color represents detected particles. The above images are representative of sampled MLN sections from **a** Air control or 0 mg/m^3^, **b** 0.06 mg/m^3^, **c** 0.2 mg/m^3^, and **d** 0.6 mg/m^3^. Blue = normal tissue. Pink = MWCNT particle deposition. White scale bar = 50 μm

**Fig. 6:**
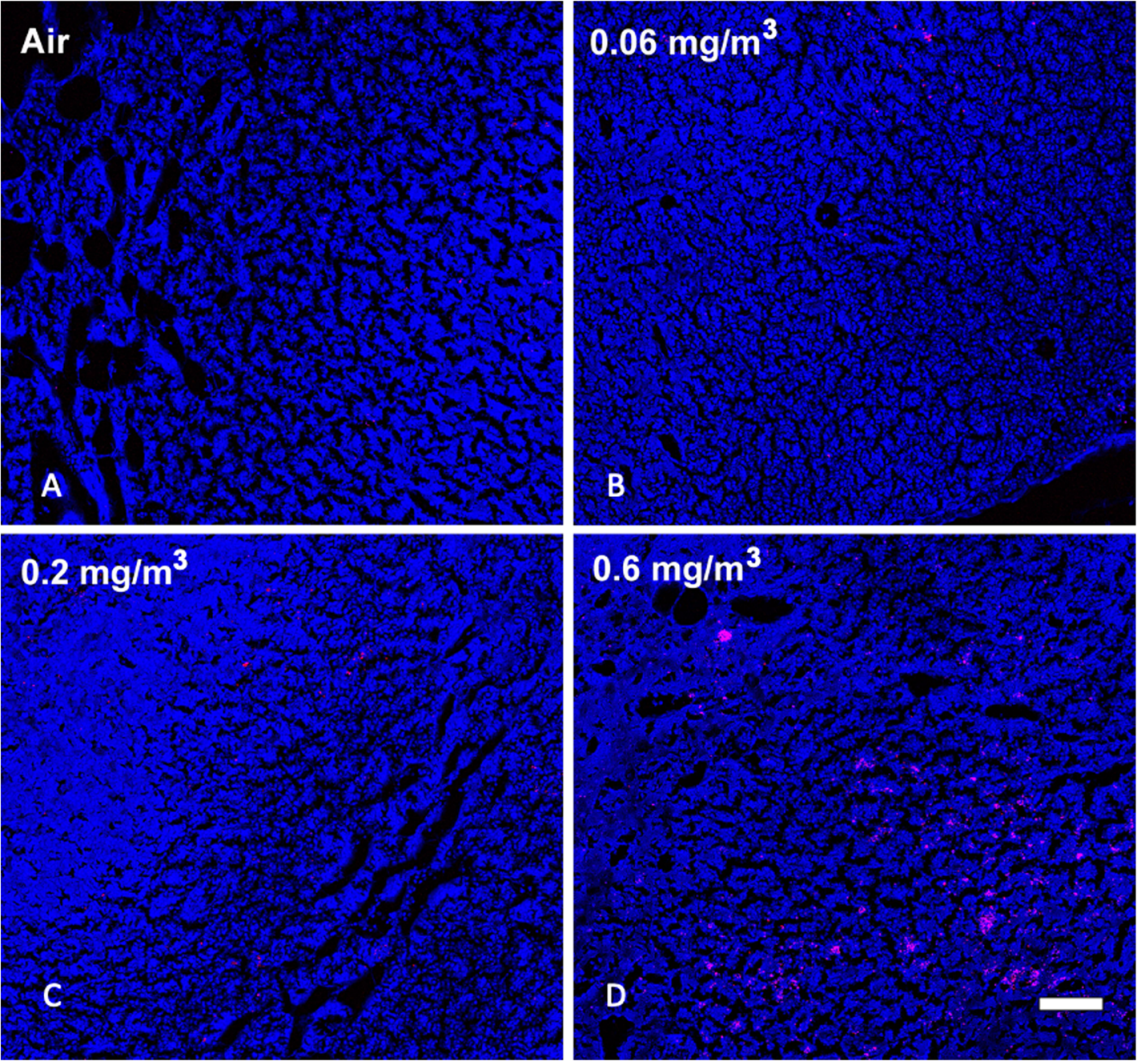
*Representative MWCNT particle deposition in brachial lymph node (BLN) tissues following 30-day exposure + 10 days recovery with all exposure groups shown.* BLN tissue was assessed for MWCNT particle deposition using stimulated raman scattering (SRS) spectroscopy, where the pink color represents detected particles. The above images are representative of sampled BLN sections from **a** Air control or 0 mg/m^3^, **b** 0.06 mg/m^3^, **c** 0.2 mg/m^3^, and **d** 0.6 mg/m^3^. Blue = normal tissue. Pink = MWCNT particle deposition. White scale bar = 50 μm

It is common for inhaled particles to be trafficked to the draining lymph nodes from the lung. We hypothesized that the aggregate MWCNT was too large for migration into the blood and most likely taken up by AM. Figure 5 illustrates the SRS results showing dose-dependent (same air control and three doses) increases in MWCNT in the mediastinal lymph nodes (MLN). There were very few particles in the lowest dose, but MWCNT were clearly evident in the two highest doses. Likewise, the brachial lymph nodes (BLN) showed MWCNT in the same two highest doses (Fig. 6). The deposition pattern was different, as there was less aggregation and more distribution in the BLN (see Fig. 6d).

### Spleen samples

#### Appearance of MWCNT in the spleen following exposures

**Fig. 7:**
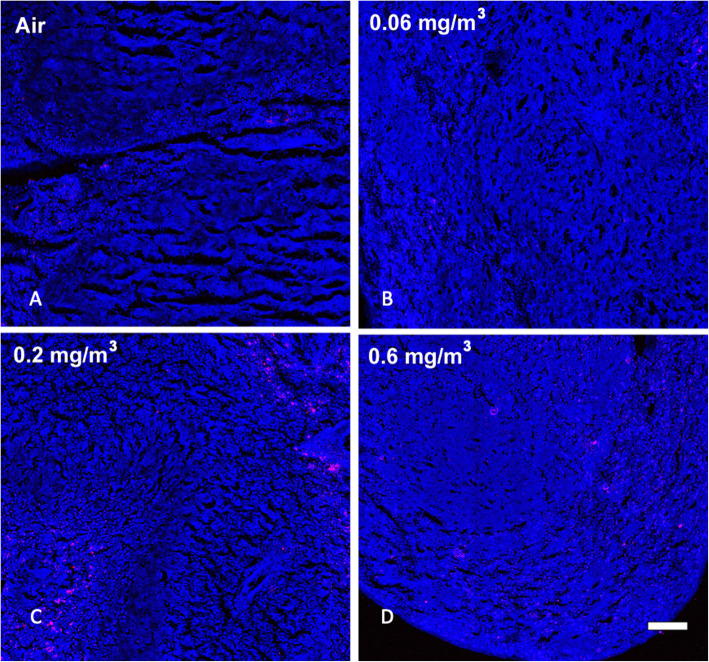
*Representative MWCNT particle deposition in spleen tissues following 30-day exposure + 10 day recovery with all exposure groups shown.* Spleen tissue was assessed for MWCNT particle deposition using stimulated Raman scattering (SRS) spectroscopy, where the pink color represents detected particles. The above images are representative of sampled lung sections from **a** Air control or 0 mg/m^3^, **b** 0.06 mg/m^3^, **c** 0.2 mg/m^3^, and **d** 0.6 mg/m^3^. Blue = normal tissue. Pink = MWCNT particle deposition

Based on previous work and published observations [4, 18], it was likely that prolonged inhalation exposures could lead to particle deposition in the spleen as a result of transport through the lymphatic system. SRS was used to examine large sections of spleen tissue for the appearance of MWCNT. Figure 7 shows regions in the spleens from control and same three particle-exposed groups of mice. A similar pattern of dose-dependent increased MWCNT was present in the spleens similar to what was observed in the lung and lymph nodes. The particle deposition was most obvious at the two highest MWCNT doses. Therefore, there was a dose-dependent deposition of MWCNT in the lungs and appearance in the lymph nodes and spleen.

#### Histological evidence of MWCNT translocation creating T cell apoptotic bodies

**Fig. 8:**
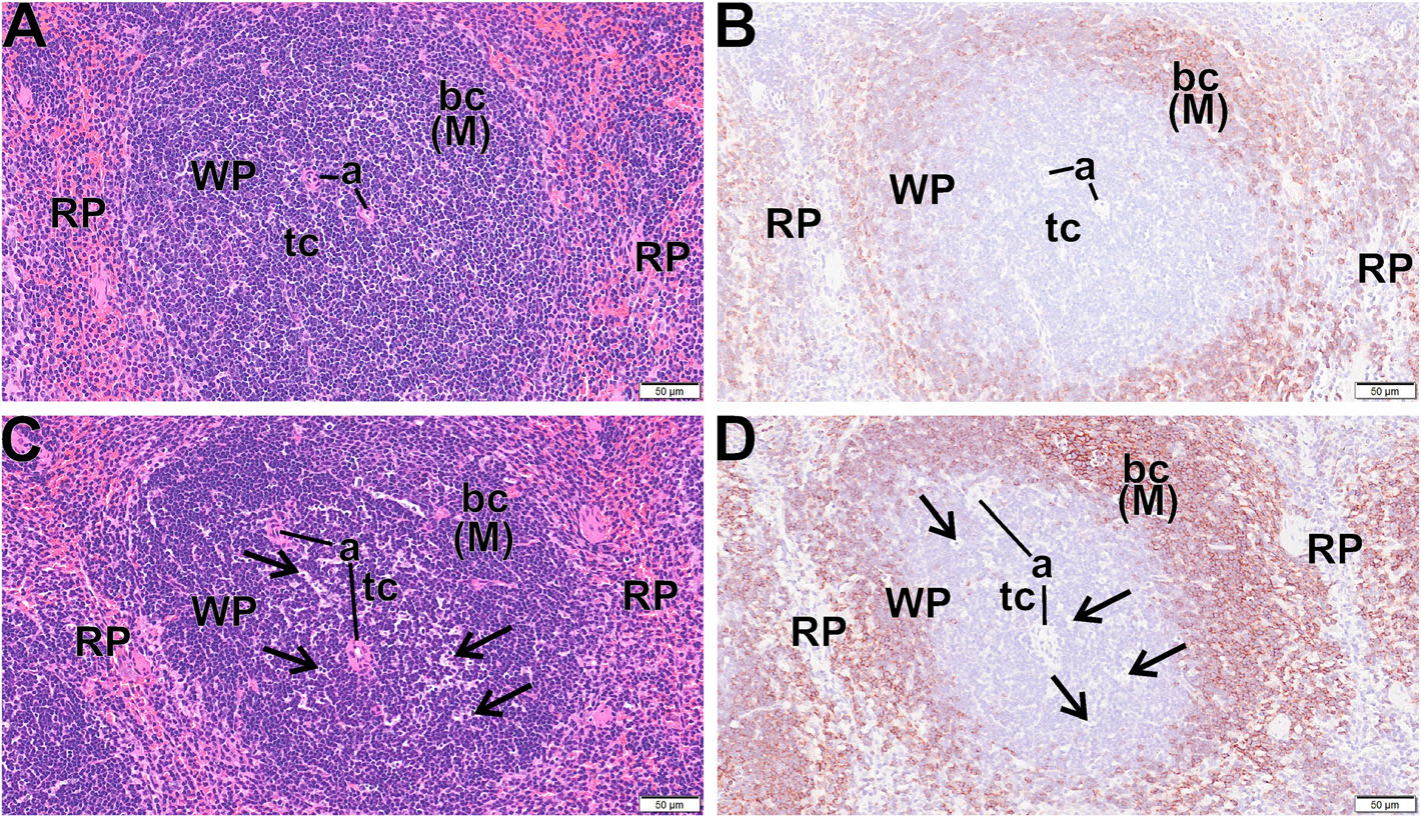
*Apoptotic bodies in spleens following 30-day MWCNT exposure + 10 days recovery.* Light photomicrographs of spleen from control mice (**a**, **b**) and exposed mice (**c**, **d**) that were histochemically stained with hematoxylin and eosin (**a**, **c**) and immunohistochemically stained for CD45R (B cells) and counter stained with hematoxylin (**d**, **d**). There are areas of apoptotic cells/bodies (arrows) in periarteriolar T cell areas of the white pulp (WP) in exposed mice but not control mice. WP – white pulp, RP - Red pulp, a – arterioles, bc (M) - B cells in mantle zone of WP

In consideration of the presence of MWCNT in the spleen, tissue sections were examined by light microscopy by a board-certified veterinary pathologist for evidence of any histopathology. For an unbiased appraisal, the pathologist had no prior knowledge of the individual exposure histories. Figure 8a - d shows light photomicrographs of spleens from control mice (Fig. 8a and b) and mice exposed at 0.6 mg/m^3^ (Fig. 8c and d) that were stained with hematoxylin and eosin (Fig. 8a and c) and additional splenic sections that were immunohistochemically stained for CD45R (B cells) and counter stained with hematoxylin (Fig. 8b and d). There were areas of apoptotic cells/bodies (arrows) in periarteriolar T cell areas of the white pulp (WP) in exposed mice, but not control mice. In addition, there appeared to be an overall decrease in the size of the white pulp region of the MWCNT-exposed mice. These findings suggested that there was some general immune-modulatory potential of the MWCNT exposure in the spleen tissues. Therefore, the activation states of the leukocytes in the spleen following all of the MWCNT doses were further examined.

#### B cells

**Fig. 9:**
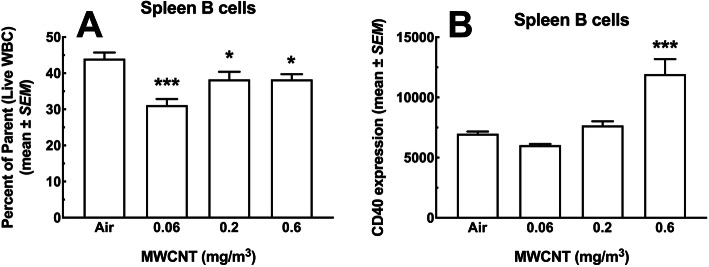
*Total leukocyte viability and B cell subpopulations in the spleen following 30-day MWCNT exposure + 10 days recovery.* **a** B cells (CD19^+^ lymphocytes) as a percent of total live WBC. Asterisks *** indicate significance at *P* < 0.001 compared to all other groups or * *P* < 0.05 compared to air control group. **b** Antigen-presenting CD40 positive B cells across exposure groups. Asterisks *** indicate significance at *P* < 0.001 compared to all other groups. All data expressed as mean ± *SEM* (*n* = 8)

The total fraction of B cells (CD19^+^ lymphocytes) from the live population of WBC is shown in Fig. 9a. There were significant decreases in B cells for every dose of MWCNT ranging from 5 to 10% cell loss. Figure 9b shows the relative CD40 expression, where CD40 is a marker of activation indicating antigen presentation activity on B cells. The high dose MWCNT (0.6 mg/m^3^) was singular in showing a significant increase (doubling) in this activation marker.

#### T cells

**Fig. 10:**
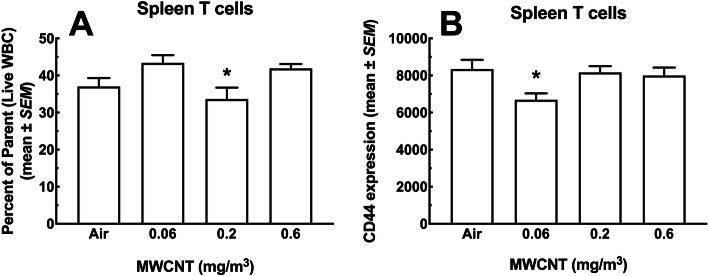
*T cell populations in the spleen following 30-day MWCNT exposure + 10 days recovery.* **a** CD3 positive T cells as a percent of total live WBC. Asterisk * indicate significance at *P* < 0.05 compared to all other MWCNT-exposed groups. **b** CD44^+^ T cells as a percent of total T cells. Asterisk * *P* < 0.05 compared to all other groups. (*n* = 8)

The percent of total T cells (CD3^+^ lymphocytes) as a fraction of total viable leukocytes is shown in Fig. 10a. There was a slight, but significant decrease in the 0.2 mg/m^3^ dose group compared to the other two doses, but not the air control. The low and high doses were not significantly different from the air control. Figure 10b shows the relative expression of the activation marker CD44 on the total viable population of T cells. Again, there was a decrease in the dose group (0.06 mg/m^3^) contrasted with the other experimental groups.

#### T cell subpopulations

**Fig. 11:**
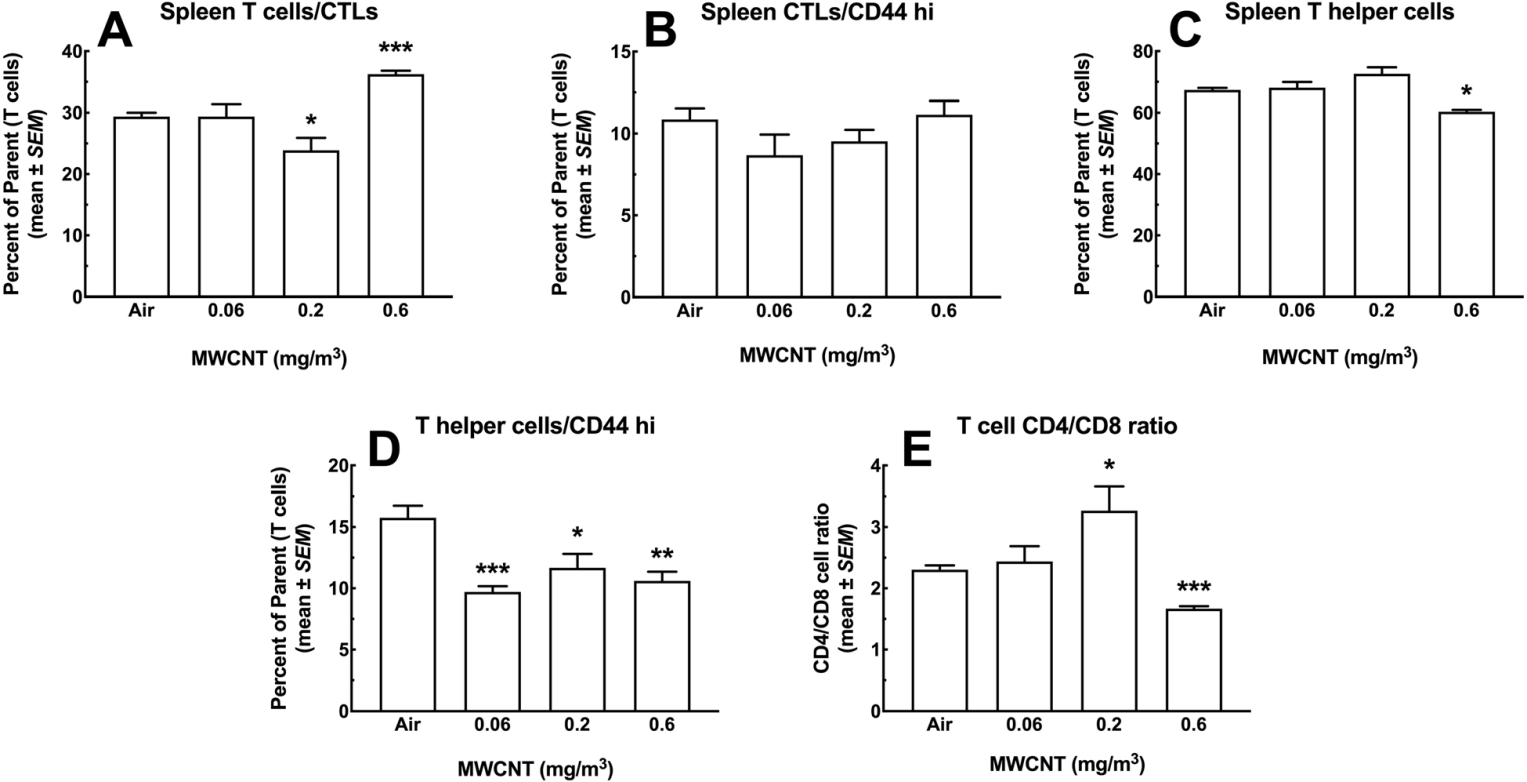
*T cell subtypes in the spleen following 30-day MWCNT exposure + 10 days recovery.* **a** CD8^+^ cytotoxic T lymphocytes (CTL) as a percent of total T cells (CD3^+^ lymphocytes). Asterisks *** indicate significance at *P* < 0.001 compared to all other groups or * *P* < 0.05 compared to air control group. **b** Activated cytotoxic T lymphocytes expressing high levels of CD44 as a percent of total T cells. **c** CD4^+^ T helper cells as a percent of total T cells. Asterisk * indicate significance at *P* < 0.05 compared to all other groups. **d** Activated T helper cells expressing high CD44 as a percent of total T cells. Asterisks *** *P* < 0.001, ** *P* < 0.01 or *P < 0.05 compared to Air control group. **e** Ratio of CD4^+^ to CD8^+^ T cells. Asterisks *** indicate significance at *P* < 0.001 compared to all other groups or * *P* < 0.05 compared to air control group. All data expressed as mean ± *SEM* (*n* = 8)

The panels in Fig. 11 show subpopulations of T cells; CD8^+^ cytotoxic T lymphocytes (CTL), CD4^+^ T helper cells, and the ratio of these two populations (CD4/CD8). Figure 11a shows the percent of CTL as a fraction of total live T cells. A slight significant decrease in the 0.2 mg/m^3^ dose group and a large significant increase in the 0.6 mg/m^3^ dose group was seen. Both differences represented about a 6% change from the air control group. In contrast, Fig. 11b shows the same CTL stained for the activation marker CD44. There were no significant deviations from the air control group or other groups for this cell expressing CD44. The T helper cells, unlike the CTL, showed some particle exposure effects. The percent of T helper cells as a fraction of viable T cells is shown in Fig. 11c. There was a significant decrease at the highest MWCNT dose relative to all other groups. Similarly, the CD44-stained T helper cells in Fig. 11d showed significant decreases in all the exposure groups compared to the air control. T helper cells activate B cells to secrete antibodies, signal macrophages to destroy ingested foreign material, and also facilitate CTL to kill defective cells. Under normal conditions the CD4/CD8 ratio in T cells should be about 2.0 [22]. Figure 9e confirms this observation in the air control group and the lowest particle dose. There was a significant increase (> 3.0) for the 0.2 mg/m^3^ dose group and a significant decrease in the CD4/CD8 ratio (< 1.6) in spleens from the 0.6 mg/m^3^ dose. The high ratio in the 0.2 mg/m^3^ dose group indicates an activated immune response. The decrease in the high dose, however, indicates immune suppression (< 1.0). Both results suggest particle-related changes in T cells, particularly the T helper cells, and show dose-dependent activation and possibly down-regulation of the immune response in the spleen, which may be related to particle burden or a result of changes to other splenic populations.

#### Monocytes and neutrophils

**Fig. 12:**
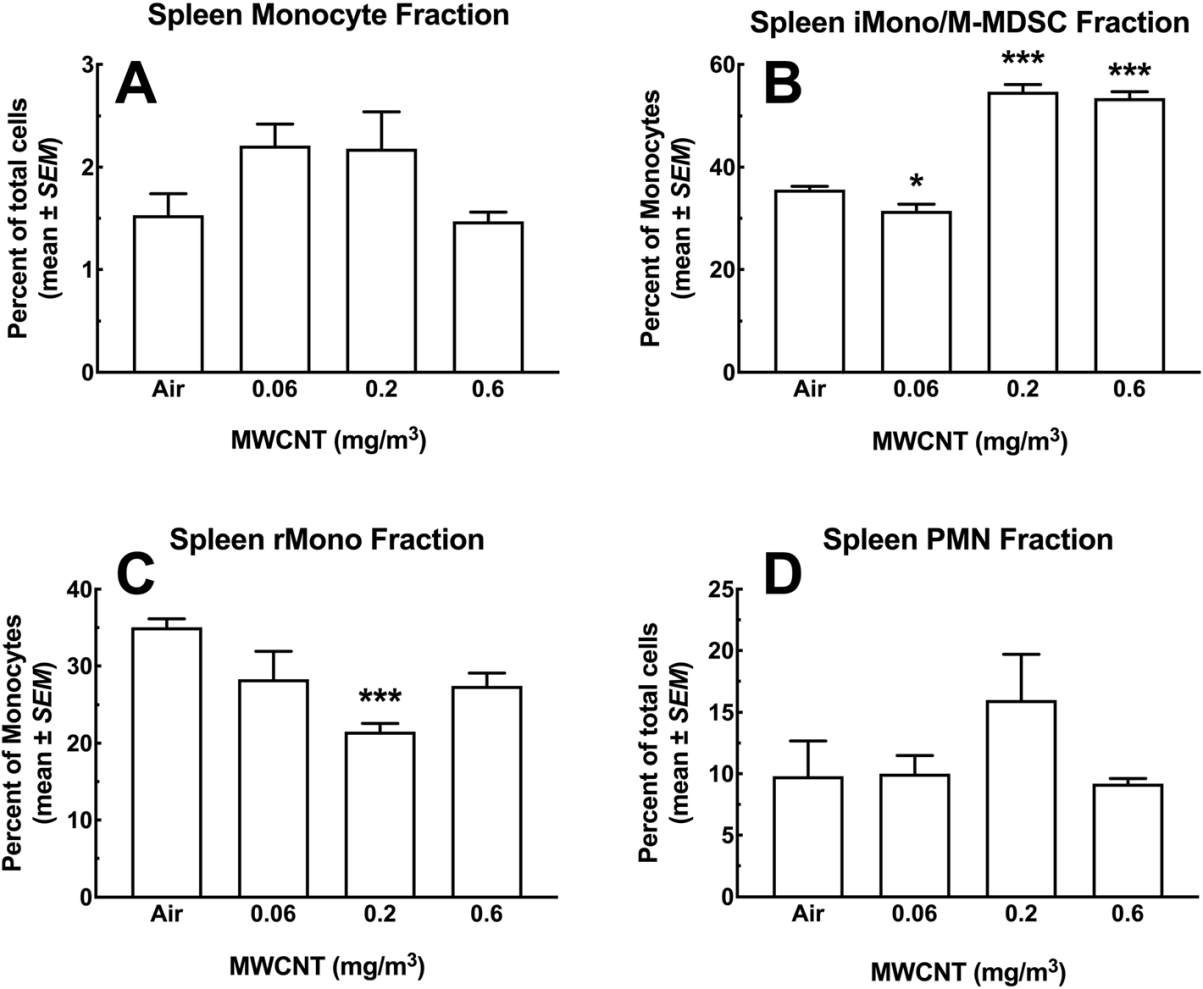
*Changes in monocyte subpopulations and neutrophil populations in the spleen following 30-day MWCNT exposure + 10 days recovery to MWCNT.* At multiple doses of particle exposure, splenic cell populations were assessed for changes using flow cytometry. Spleens were removed and disrupted to achieve single-cell suspensions for analysis. **a** Change in total monocyte populations (CD11b^+^ Ly6C^lo/hi^), where a nonsignificant increase in the total monocyte population were observed. **b** Shows changes in the inflammatory monocyte populations with significant increases in iMono/M-MDSC (CD11b^+^ Ly6C^hi^) for the highest 2 doses compared the low dose and the air control *** *P* < 0.001. **c** Shows changes in the resident monocyte populations with nonsignificant and significant decreases in rMono (CD11b^+^ Ly6C^lo^) for all particle doses compared to the air control *** *P* < 0.001. **d** Neutrophil (PMN, CD11b^+^ Ly6G^+^) percent showed no significant changes across dose, but there was a nonsignificant increase in the 0.2 mg/m^3^ dose. Mono = total monocytes; iMono = inflammatory monocytes; M-MDSC = monocytic myeloid-derived suppressor cells; rMono = resident monocytes, and PMN = neutrophils. All data expressed as mean ± *SEM* (*n* = 8)

After gating removal of neutrophils, monocytes were classified as CD11b^+^ Ly6C^lo/hi^, and distinguished from macrophages based on lack of MHC II expression. Figure 12a shows the total percent of monocytes in the spleen as a fraction of viable WBC. The monocyte percentages were low varying from 1.5 to 2.2% depending on the exposure group. Comparisons between different groups showed no significant differences in the monocyte levels. Unlike the total monocyte percentages, there were significant changes in the CD11b^+^ Ly6C^hi^ population as a fraction of the total monocyte count. This phenotype has been associated with either the inflammatory monocyte (iMono) or the monocytic myeloid-derived suppressor cell (M-MDSC) [23, 24]. Figure 12b shows this data where there was a slight, but significant decrease in this population for the lowest MWCNT dose compared to the air control group. In contrast, there was a very large significant increase (roughly 2x) in iMono/M-MDSC for the two highest MWCNT doses compared to both air control and the low dose groups. The resident monocyte, (rMono) CD11b^+^ Ly6C^lo^ population shown in Fig. 12c showed decreases in this monocyte subtype for all exposure groups with the 0.2 mg/m^3^ being significant compared to air control. The spleen neutrophils (CD11b^+^ Ly6G^+^) at around 10% did not change significantly across the exposure groups (Fig. 12d).

#### Macrophages

**Fig. 13:**
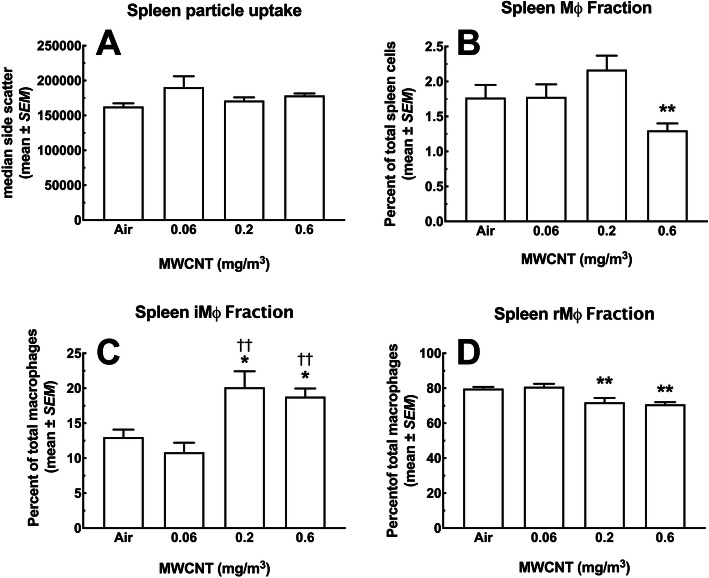
*Changes in macrophage subpopulations in the spleen following 30-day MWCNT exposure + 10 days recovery to MWCNT.* At multiple doses of particle exposure, splenic cell populations were assessed for changes using flow cytometry. Spleens were removed and disrupted to achieve single-cell suspensions for analysis. **a** General particle uptake (presumably by Mφ) measured by side-scatter showed no change across the dose-range compared to Air control. **b** This graph shows the percent Μφ (CD11b^+^ MHCII^mid/hi^ Ly6C^lo/hi^) in the spleen, which decreases significantly at the highest dose ** *P* < 0.01 compared to all other groups. **c** This panel shows changes in the inflammatory macrophage subpopulation with significant increases in iMφ (CD11b^+^ Ly6C^hi^) for the highest 2 doses compared the low dose †† *P* < 0.01 and the Air control * *P* < 0.05. **d** Shows changes in the resident macrophage subpopulation with significant decreases in rMφ (CD11b^+^ Ly6C^lo^) for the two highest doses compared to the lowest dose and the air control ** *P* < 0.01. Mφ = total macrophages; iMφ = inflammatory macrophages; rMφ = resident macrophages. All data expressed as mean ± *SEM* (*n* = 8)

MWCNT particle intake, measured by side scatter for the spleen CD11b^+^ MHCII^mid/hi^ Ly6C^lo/hi^ macrophages, is represented in Fig. 13a. There were no significant differences in this metric. Figure 13b shows the percent of spleen macrophages as a fraction of total viable WBC. A significant decrease in the macrophage percent at the highest dose was seen, compared to all other groups. The decrease was about 0.5% relative to control, as the total macrophage percent was a relatively small fraction of total WBC (~ 1.75%). In contrast, the fraction of inflammatory macrophages (iMΦ) CD11b^+^ Ly6C^hi^, almost doubled in the two highest dose groups compared to air control and the low dose MWCNT groups (Fig. 13c). Similarly, the resident macrophages (rMΦ), CD11b^+^ Ly6C^lo^, showed a corresponding significant decrease at the two high-particle doses (Fig. 11d). The resident macrophages represented most of the spleen macrophage fraction (~ 80%), and showed a (~ 10%) reduction. Whereas, the inflammatory macrophages represented only about 13% of the total macrophages with a less than 10% increase for the two high doses.

#### Inflammatory gene changes in the spleen

**Fig. 14:**
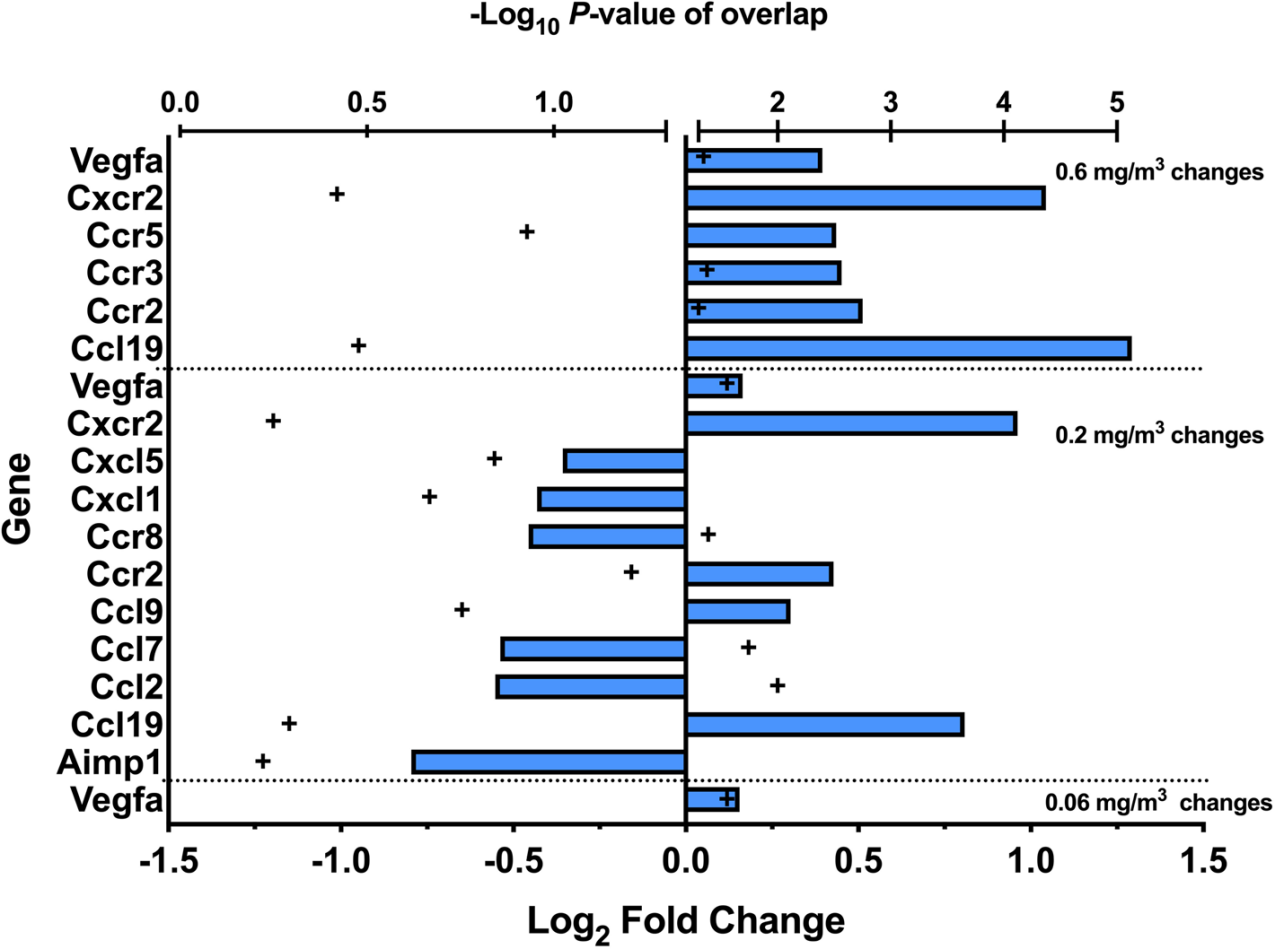
*Inflammatory mediator genes changes in the spleens following 30-day MWCNT exposure + 10 days recovery.* Direction and magnitude of the fold change (> 2-fold untransformed) versus ‘air’ control is shown by the blue bars relative to the Log_2_ fold change scale. Positive values are increases and negative values are decreases in genes from MWCNT-exposed versus air control. Statistical significance at *P* < 0.05 is indicated by + symbols greater than 1.5 -Log_10_ *P*-value or all + symbols in the right hemisphere of the graph. (*n* = 8)

To gain a more extensive description of changes in the spleen, a gene array analysis was conducted. Figure 14 is a transformed double X plot showing the genes that changed (increased or decreased) more than 2x and the *P* values associated with that change. Trends in the data showed that the low dose MWCNT exposure produced a significant change in Vegfa compared to the air control. Additionally, the middle dose group (0.2 mg/m^3^) had roughly the same number of decreased gene expression as increased gene expression in contrast to the high dose where the changes in gene expression were all increased. Lastly, there were several genes that showed up in more than one dose/control comparison. Vegfa, Cxcr2, Ccr2, and Ccl19 appear in at least two of the dose/control contrasts, suggesting their contribution/importance in mechanistic pathways. See Supplemental Table S4 for a comprehensive list of detected changes.

#### Cytokine and Cathepsin levels in the blood plasma

**Fig. 15:**
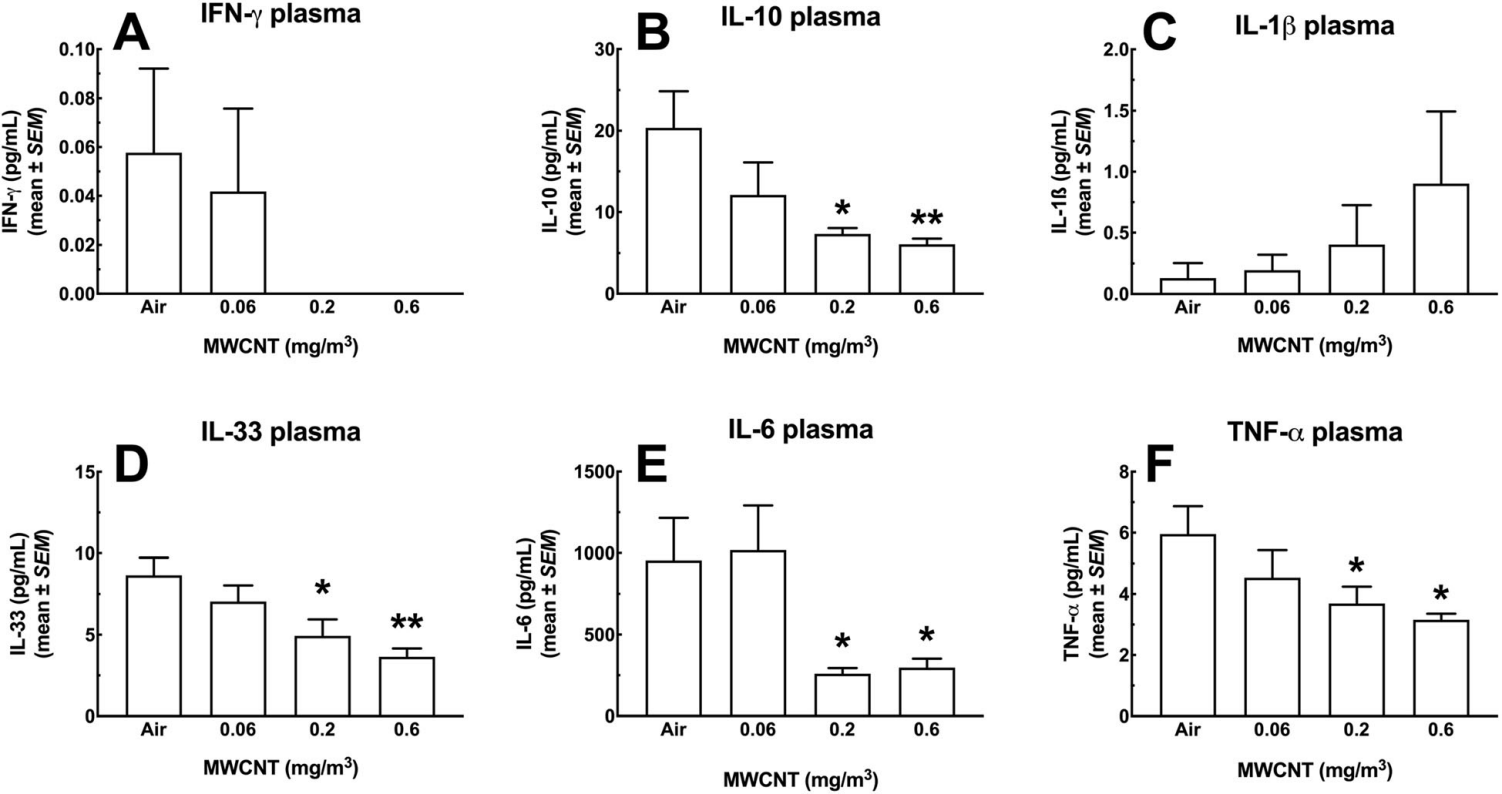
*Cytokine changes in the blood plasma of mice following 30-day MWCNT exposure + 10 days recovery.* Heparinized blood was used to assess a panel of analytes. The MesoScale Discovery (MSD) system was used to assay for multiple analytes in a single sample. In the figure above, there was a significant observed increase in several inflammatory mediators including **a** IFN-γ, **b** IL-10, and **c** IL-1β, **d** IL-33, **e** IL-6, and **f** TNF-α. Asterisks * indicate statistical significance at *P* < 0.05, or ** where *P* < 0.01 or *** where *P* < 0.001 compared to corresponding ‘air’ control by Holm-Sydak *post-hoc* analyses. All data expressed as mean ± *SEM* (*n* = 8)

**Fig. 16:**
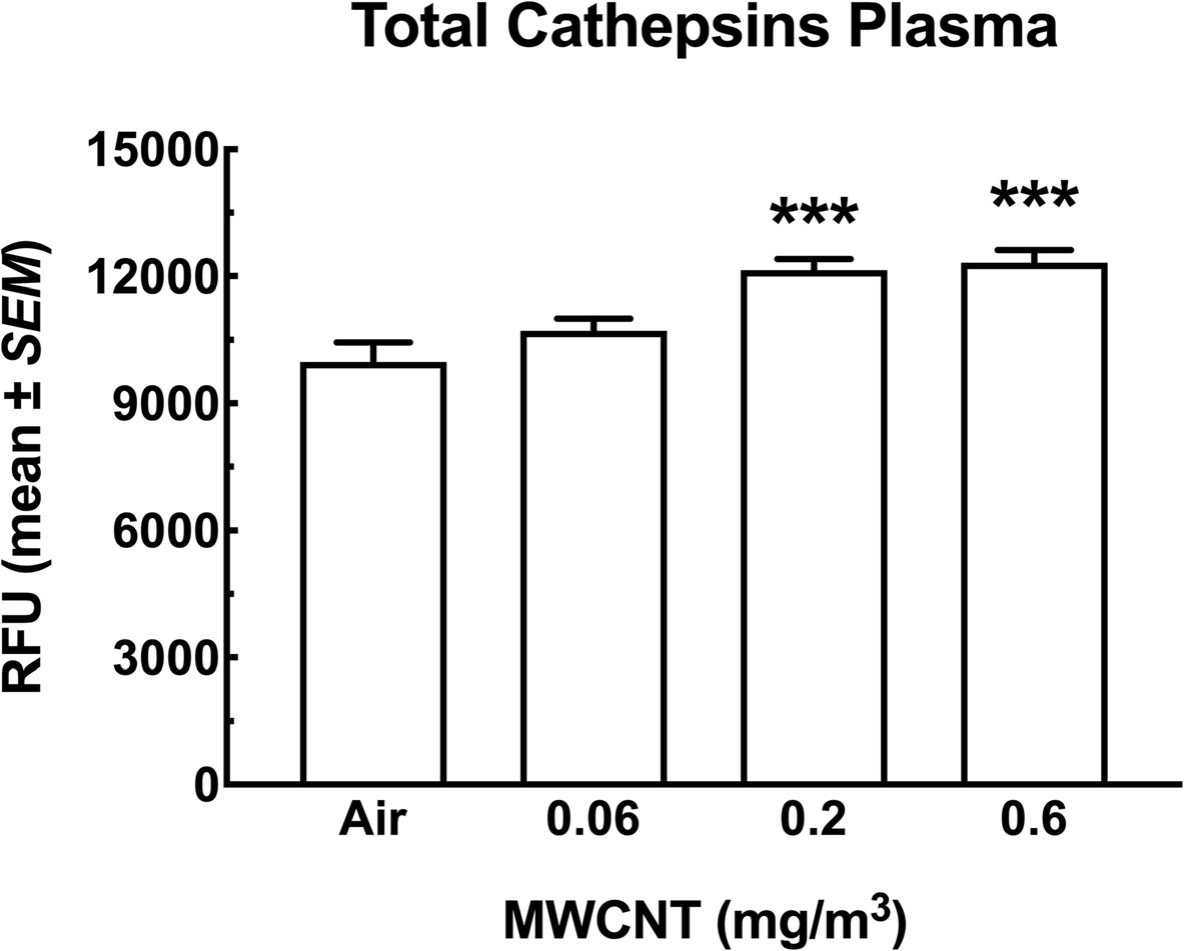
*Changes for total cathepsins in the blood plasma of mice following 30-day MWCNT exposure + 10 days recovery*. Mean ± *SEM* relative fluorescence units representing total cathepsin levels in the blood plasma following MWCNT exposure or sham. Asterisks *** indicate significance at *P* < 0.001 compared to the air control and 0.06 mg/m^3^ exposure groups. All data expressed as mean ± *SEM* (*n* = 8)

In order to evaluate the systemic implications of the changes in the lungs and spleen, levels of a number of inflammatory cytokines were measured in the circulation. Figure 15 shows six cytokines measured in the blood plasma in this study. Five of the six cytokines (IFN-γ, IL-10, IL-33, IL-6 and TNF-α) showed large and mostly significant dose-dependent decreases in plasma levels (Fig. 15a, b, d, e and f). Only IL-1β showed a dose-dependent increase (Fig. 15c). Figure 16 shows a dose-dependent increase in total cathepsins that was significant for the two highest MWCNT exposure groups.

#### MWCNT were not detected in kidney or liver

**Fig. 17:**
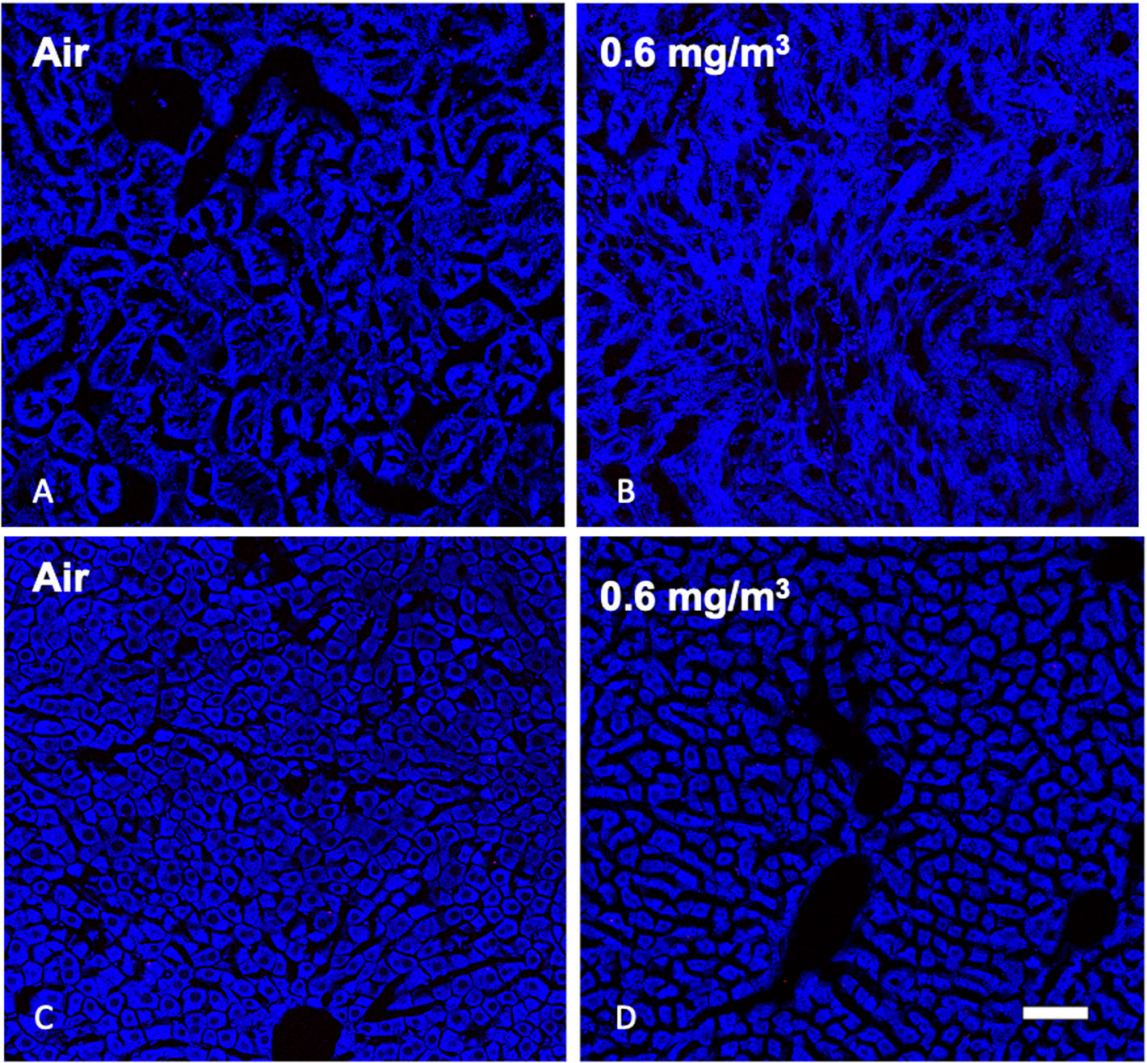
*Representative MWCNT particle deposition in kidney and liver tissues following 30-day exposure + 10 days recovery with zero and high exposure groups shown.* Lung tissue was assessed for MWCNT particle deposition using stimulated raman scattering (SRS) spectroscopy, where the pink color represents detected particles. The above images are representative of sampled kidney sections from **a** Air control or 0 mg/m^3^, **b** 0.6 mg/m^3^, and liver sections **c** Air control or 0 mg/m^3^, and **d** 0.6 mg/m^3^. Blue = normal tissue. Pink = MWCNT particle deposition. White scale bar = 50 μm

In order to examine the possibility that the MWCNT particles were transported in the blood or ingested during exposures we assessed particle deposition in the kidneys and liver respectively. Figure 17a is a kidney section from the air control and Fig. 17b shows a kidney from the high dose exposure group. There were no particles found in any of the kidney sections examined. Similarly, examination of the liver showed no particles in the tissue sections examined. Figures 17c is the air control liver sections and Fig. 17d is the high dose liver sections. The final organ examined was the OB and there were particles observed in all particle exposure groups with the largest deposition in the highest dose (Supplementary Figure S2).

## Discussion

Exposure to MWCNT is an ongoing concern and challenge with respect to toxicological evaluation. The majority of MWCNT exposures take the form of inhalation, ingestion, or topical, and as these types of exposures are increasing an understanding of potential health implications is essential. The present study evaluated potential local and systemic effects following chronic inhalation exposure to MWCNT by analyzing particle deposition, translocation, inflammatory parameters, cell immune cell population changes, and tissue pathology.

Previous studies utilized in vitro assays to categorize MWCNT as “bioactive” or “benign” based on their ability to induce cytotoxicity and/or promote secretion of inflammatory mediators (i.e. IL-1β) [9, 25]. Those studies demonstrated that the particles used in the present work were comparatively benign based on the relative inactivity of the MWCNT in cell culture studies. The in vitro studies attempted to model/predict the outcomes of inhalation exposure based on incubation with alveolar macrophages using the THP-1 culture model using endpoints of cytotoxicity and inflammasome activation via IL-1β production [8]. The present study utilized these benign MWCNT in an effort to either confirm the benign designation or determine unforeseen systemic effects. While the data from the present work suggests that these MWCNT caused minimal lasting effects in the lung, there were distal effects in immune tissue that may result in significant alterations to the immune status of an exposed individual.

Particles designated as “benign” using in vitro screening assays are predicted to have little in vivo biological activity. Generally, biological activity in these studies refers to either cytotoxicity and/or cell activation/inflammation [25], and would be predicted to have limited inflammatory or fibrotic effects in the lungs. However, little is known or understood about systemic or long-term effects. In the present study, we found scarce evidence of effects in the lungs (no significant population changes and small increases in cytokines (Figs. 2 & 3), although lung macrophages appeared to phagocytose the particles in a dose-dependent mechanism (Fig. 2a). In assessing potential distal effects, MWCNT were found in lymphoid organs including spleen (Fig. 7) and lymph nodes (Figs. 5 and 6), but not the liver or kidneys (Fig. 17). These observations suggest that potential distal effects of “benign” MWCNT were potentially mediated by the immune system, wherein local macrophages trafficked the particles through normal immune pathways. Additionally, while studies evaluating splenic deposition of nanoparticles [26, 27], noted particles introduced intravenously or intraperitoneally were found in the red pulp regions, the present studies suggest deposition in the white pulp areas with resulting immune effects of population changes and apoptosis. The end result of this particle translocation was terminal deposition in the spleen resulting in more substantial chronic effects.

Potential chronic effects were suggested from evidence provided from observations of tissues distal to the site of exposure. While particles were detected in several tissues, only the spleen was able to be assessed by flow cytometry for cell changes. Cell population changes in the spleen demonstrated increases in inflammatory cells (Figs. 12 and 13), laying the groundwork for alterations in subsequent immune responses. While functional studies of splenic cell populations were not performed, future investigations in assessing the long-term systemic health effects of MWCNT exposures is warranted. In addition, multiple apoptotic bodies were visualized in splenic tissue following the MWCNT exposures (Fig. 8), providing additional evidence of alterations to the spleen, that may have long term immune implications to the host. We propose that the higher exposures of MWCNT resulted in increased translocation, splenic alterations, and observed distal changes (LN deposition, splenic toxicity, and systemic mediators), despite relatively minor lung (local) effects (Figs. 3 and 4). While the delivery of the MWCNT-loaded cells to the spleen appears to alter the immune cell populations which eventually result in an altered immune status, additional studies will need to expand these assessments to regional lymph nodes to better define this effect. This new, altered immune status, observed here as an increase in inflammatory macrophages and monocytes, could have significant consequences on a variety of outcomes including, but not limited to autoimmune disease, vaccination responses, or fibrosis development.

Several observations in the present study suggest an effect on influential myeloid populations in the spleen. Monocytes are generally considered blood cells that “roam” systemically before translocation to tissues and maturing to macrophages. Recent studies have reported a “reservoir” of immature, heterogeneous monocytic population for responses to inflammatory events [23, 28]. The increased CD11b^+^Ly6C^+^ splenic population (Fig. 12b) have been categorized in the literature as one of two myeloid-derived cells that could be constituents of this reservoir [23, 28–33]. While the same surface markers have been found on either inflammatory monocytes (iMono) or monocytic myeloid-derived suppressor cells (M-MDSC), the current literature suggests that these cells are closely related, if not actual counterparts [32]. The M-MDSC are considered to possess anti-inflammatory/immune suppressive properties that have been extensively described in tumor models [34–37], while the iMono are categorized as inflammatory [23]. Some data suggest that these two cell populations may be points on a transition scale [28]. Due to the overlapping of surface markers, some investigators have proposed categorizing these populations by including additional phenotypic markers (i.e. cytokines) and functional assays. Bronte, et al., proposed an algorithm and even included the term MDSC-like cells (MDSC-LC) to describe what appear to be MDSC, but do not possess suppressive activity [29]. The M-MDSC have been found to traffic to sites of inflammation in a variety of models including tumors, ischemic myocardial injury, ozone inhalation, and silica exposure [23, 34, 38–41]. In addition, the present data has shown an increase in CCR2, a chemokine receptor involved in monocytic cell recruitment, which the literature has shown to be more of a M-MDSC marker than the iMono, and key to the translocation. The data show that these cells could provide a mechanism of inflammation regulation, that can contribute to pathology (i.e. TGFβ) [23, 38], suggesting the profound effect the cells are able to impart in different situations. We also noted apoptotic bodies in the T cell regions of the spleen (Fig. 8), suggesting a potential effect, possibly via reactive oxygen species (ROS), of this increase in M-MDSC/iMono population [31, 35], where Ohl, et al., described this M-MDSC function in decreasing effector T cells in their model [31] and Beury et al., described both the production of ROS by MDSC and its ability to survive the oxidative burst, via the function of Nfr2. Further work in this area will need to be done to confirm this potential role. Additionally, two genes, Vegfa and Ccr2, were consistently increased in the present model, and have both been shown to be associated with MDSC populations [28, 42, 43]. Ccr2 is key to trafficking of these cells and Vegfa has been described in the accumulation of MDSC, as well as being produced by these cells in certain models. Our observed changes, based on the type of cells involved, whether the inflammatory iMono or the immunosuppressive M-MDSC, may have significant effects on host immune responses and suggest a potential for unanticipated outcomes with subsequent exposures or insults.

Adverse effects resulting from pulmonary exposures to particles/nanoparticles include a variety of outcomes. Depending on the type of nanoparticle, mice have presented with inflammation [11, 25], fibrosis [11, 44], as well as extrapulmonary effects (i.e. autoimmune exacerbation) [45]. Generally, severe adverse effects have been predicted with in vitro models. In these cell culture models, particles that induce cytotoxicity, inflammation, or inflammasome activation have resulted in observed pathology in exposed animals [11, 25]. In a companion study of the present work, the MWCNT appear to decrease the Th2 response in a house dust mite model (HDM), as well as increase inflammation and fibrosis [20]. While the MWCNT exposure alone did not result in significant lung pathology in our animals or the Ihrie et al., study [20], their results suggest an immunotoxic, or immunopotentiation effect that altered the immune response in the HDM model. In addition, the study (Ihrie et al) noted increased levels of TGFβ, a known promoter of lung fibrosis [46], in the lavage fluid of mice. These observations illustrate the potential contribution of M-MDSC, as a producer of TGFβ, to the pathology in the HDM model and may suggest that the effect of the original MWCNT exposure is indirect via the activity of a potent immune mediator. Additionally, M-MDSC have been described as contributors in multiple cancers [31, 33, 34, 42, 47]. While tumor biology was not a consideration for the present work, others have hypothesized a role for certain bioactive (defined by in vitro assays) nanoparticles to possess cancer-inducing properties [48]. In contrast the effects observed in our study of these “benign” (in vitro-defined) MWCNT on MDSC populations suggests another potential downstream effect of these MWCNT with respect to cancer. Further work on the contribution of this splenic population on systemic pathology will need to be performed to confirm this model.

## Conclusion

The present study suggests the need for more extensive assessments of nanomaterial exposures, evaluating both short- and long-term effects. While in vitro assays (cell cultures, cytokine profiles, cytotoxicity) may be informative, our observations suggest that the translation of the in vitro characterization to in vivo effects may not always be direct. With the deposition of the pulmonary-exposed MWCNT in extrapulmonary immune tissues (spleen), in addition to alterations in splenic populations, there are unanticipated consequences with these “benign” particles. Additionally, in light of the populations that appear to be affected (M-MDSC/iMono), concern for systemic effects by these regulatory/inflammatory cell populations is merited. This result is supported by the collaborative effort using the HDM model [20]. Based on our results, we propose that while in vitro cytotoxic/bioactive MWCNT may translate to predicted, and observed, localized effects (i.e. inflammation, fibrosis, toxicity), those MWCNT characterized as “benign” or lacking bioactivity in cell culture assays could have significant systemic effects in vivo, despite that lack of local pathology.

## Methods

### Mice

Male B6C3F1 mice were used in this study, originating from Taconic, Columbus, OH. Health screening testing were provided prior to shipment. All mice were maintained in pathogen-free conditions (22 ± 2 °C, 30–40% humidity, 12 h light/12 h dark cycles) and offered food and water ad libitum in the animal facilities of Battelle Laboratory, UC Davis, California, and after arrival at the University of Montana (UM, Missoula, MT). Mice were sacrificed within 1 day of arrival at the University of Montana. All experiments met the approval of the Institutional Animal Care and Use Committee (IACUC) of each respective site.

### Particle characterization and nanoparticle challenge

The particle chosen for the study was selected because of high purity, and low amount of residual metal (in particular, Ni, Y and Fe) catalyst. The MWCNT was obtained at Sun Innovations, Fremont, CA). See Table 1 for a detailed description. Further characterizations, including SEM of particle can be found elsewhere [19, 20]. Whole body exposure was performed by Battelle Laboratory (Ohio, USA). Male B6C3F1 mice between ages five and 6 weeks of age were exposed to the MWCNT by whole body exposure for 30 days (weekdays only, total 22 days), 6 h/day. Eighty mice were exposed to MWCNT with twenty in each group at the following doses: 0 mg/m^3^ (control), 0.06 mg/m^3^, 0.2 mg/m^3^, and 0.6 mg/m^3^. A most detailed description of the mouse exposure protocol can be found here: [19]. The day after the last exposure, mice were transported to the University of California-Davis (California) where they were divided into equal groups. Seven days later, half (forty) of the mice were shipped via air and ground transportation to UM, where mice were harvested with 24 h.

**Table 1:** MWCNT Characterization [19]

Manufacturer	Sun Innovations
Average Diameter^a^	15 nm (*N* = 90)
Length Estimate^b^	2.6 um (*N* = 102)
Purity^c^	99%
Elemental Analysis^d^	
C	97%
H, N, S	< 0.5%
Ni	0.52%
Cl, Co, Fe, Cu	< 0.01%
Surface Composition^e^	
C	98%
O	2%
Average Surface Area^f^	175m^2^/g
Skeletal Density^g^	2.0 g/cm^3^
Zeta potential^h^	−30 mV

^a^*TEM* Transmission electron microscopy

^b^*SEM* Scanning electron microscopy

^c^*TGA* Thermogravimetric analysis

^d^Carbon, hydrogen, nitrogen and sulfur determined by C,H,N,S analyzer. Ni presence indicated by EDS spectra

Ni, Cl, Co, Fe and Cu quantitated by NAA

^e^XPS survey scan

^f^BET analysis

^g^Helium gas picnometry

^h^Electrophoretic velocimetry

### Tissue collection

At harvest, mice were euthanized via intraperitoneal injection of 0.1 mL sodium pentobarbital (Euthasol™). Lungs of the first eight mice from each group of ten were surgically removed and lung lavage fluid (LLF) collected prior to fixation. The spleens of these mice were surgically removed and prepared as a single cell suspension for flow cytometry. The lungs, spleens, kidneys, liver, mediastinal lymph nodes (MLN), brachial lymph nodes (BLN) and olfactory bulb (OB) from the remaining two mice from each group were surgically removed and fixed in 1 mL 4% paraformaldehyde-phosphate buffered saline (PFA) overnight at 4 °C. In addition, heparinized blood was collected from the heart, centrifuged, and plasma removed and frozen (− 20 °C) for cytokine measurements.

### Lung lavage

For lung lavage, one mL of ice-cold sterile saline was instilled and withdrawn through a tracheotomy tube and repeated three times. The fluid was then put into an eppendorf tube, centrifuged at 400 x *g* for 5 min at 4 °C, supernatant withdrawn and placed in a clean labeled tube, which was then frozen at − 20 °C for future cytokine measurements. Half of the original cell-pellet was used for the cell count and differential. Lung lavage cells were isolated by centrifugation (400 x *g*, 5 min, 4 °C), resuspended in PBS and cell counts (40 μL used) obtained using a Coulter Z2 particle counter (Beckman Coulter, Miami, FL). Cytospin preparations (Thermo Shandon Limited, Cheshire, England) were performed on approximately 5 × 10^4^ cells for staining the cells, using a HemaTek 2000 automated staining system (Bayer HealthCare LLC, Terrytown NY), which is comparable to the Wright–Giemsa protocol. The other half of the individual cell-pellets were combined from two mice and analyzed by flow cytometry.

### Spleen single cell preparation

At harvest, the spleens of the first eight mice from each group of ten were surgically removed and prepared as a single cell suspension. Briefly, spleens were pushed through a tissue-strainer (Falcon Ref # 352350), washed with sterile PBS, lysed with NH_4_Cl to remove red blood cells, and resuspended in staining buffer (PBS with 1% BSA and 0.1% sodium azide) at a concentration of 10 × 10^6^/mL.

### Flow Cytometry

For spleens, staining was done using 100 μL of cell suspension (1 × 10^6^ cells) which were blocked with Fc receptor block (Anti-CD16/32 Tonbo Biosciences, San Diego, CA) to reduce non-specific antibody binding. The panels of antibodies used included: CD45 BV510 (clone 30-F11), CD19 PE-Dzl 594 (clone 6D5), CD40 PE-Cy7 (clone 3/23), Ly6C PE-Dzl 594 (clone HK1.4), Anti-Ly6G AF 700 (Clone 1A8) all from BioLegend (San Diego, CA); CD4 FITC (clone RM4–5), CD8a (clone 53–6.7), CD44 (clone IM7), CD3 APC (clone 17A2), MHC II FITC (clone M5/114.15.2), CD11b PerCP-Cy5.5 (clone M1/70), F4/80 PE (clone BM8.1), CD11c APC (clone N418) all from Tonbo Biosciences, San Diego, CA; and Siglec F APC-Cy7 (clone E50–2440) from BD BioSciences, San Diego, CA. Dead cells were excluded using 4,6-diamidino-2-phenylindole dihydrochloride (DAPI) (Molecular Probes/Invitrogen). Approximately 5 × 10^4^ cells from the LLF were stained in 100 μL staining buffer, following blocking with FC receptor block as above. Only the myeloid markers from the antibodies listed above were used due to cell numbers. Flow cytometry was performed using an Attune NxT Acoustic Focusing Flow Cytometer (Life Technologies) and data analyzed using FlowJo software v.10.0 (FlowJo LLC, Ashland, OR). See Supplemental Table S1 (Myeloid population surface markers), Supplemental Table S2 (Lymphoid population surface markers), and Supplemental Table S3 (Flow Cytometry reagents/Titrations) for more detail including instrument specifications, antibody titrations, and phenotypic descriptions.

### Histological preparation

Organs from two mice from each group were collected for histology. This included whole lungs, spleen, brachial and mediastinal lymph nodes, kidneys, liver and olfactory bulb. At harvest, tissue was fixed overnight in 4% paraformaldehyde-phosphate buffered saline (PFA), then rinsed the following day, placed into labeled cassettes and submerged in ethanol until processed. Post fixation, the tissues were processed in tissue specific programs in a Leica ASP300 tissue processor (Buffalo Grove, IL) using Xylene as the clearing agent and Paraffin Extra embedding medium with vacuum applied in the paraffin baths. Tissue sections (5 μm thickness) were cut on a Leica 2235 rotary microtome. All slides were stained with Hematoxylin and Eosin (H & E) in a Leica 5010 auto-stainer. For the H&E program, Mayer’s Hematoxylin and Alcoholic Eosin Y were used from Cancer Diagnostics (Durham, NC). H & E slides were imaged on a Nikon Eclipse 800 microscope (Melville, NY) using an Olympus DP71 camera and cellSens software, V4.2 (Center Valley, PA). Immunohistochemistry staining of CD3^+^(T-cells) and CD45R^+^(B-cells) was done on the spleens at the Michigan State University Histopathology Laboratory. A board-certified veterinary pathologist (JRH) scored and evaluated the immunohistochemical (IHC) results (see results section for more details). Low magnification H & E-stained control and high dose MWCNT tissue examples can be found in Supplemental Figure S3.

### Stimulated Raman scattering

Unstained/uncovered slides were prepared and sent to the Beckman Institute, University of Illinois at Urbana-Champaign (SD and RB) for Raman imaging using Stimulated Raman Scattering (SRS) to visualize the MWCNT in a similar manner as previously described [10]. The images of unstained lung tissue sections were acquired using a SRS imaging setup built in house, which is based on a two-photon laser scanning microscopy system [49]. The SRS microscope is integrated with a dual-output (1064 nm/532 nm, 80 MHz) ultrafast oscillator (Lumera, Coherent Germany) coupled into an optical parametric oscillator (OPO) (Levante Emerald, APE Germany) to provide tunable (750 nm – 970 nm) ~ 6 ps pulse trains. The 1064 nm output from the oscillator is used as the Stokes beam and the output from the OPO is used as the pump beam, which is tuned to match a Raman mode of interest. The Stokes pulse train (1064 nm) is amplitude-modulated at 7 MHz by an electro-optic modulator (EOM, Conoptics) and beams are spatiotemporally overlapped and sent collinearly to the SRS microscope. The images were acquired using a 50x (0.95 NA, Zeiss) objective and a custom-built large area photodiode (PS100–6, First sensor) detector. A high OD band-pass filter (Chroma Technology, 890/220 m) was used to selectively transmit the pump beam and to block the Stokes beam for stimulated Raman Loss (SRL) detection [50]. The SRL signals (based on transferred modulation from the Stokes beam to the pump beam), thus acquired, were demodulated and amplified by a lock-in amplifier (HF2LI, Zurich Instruments) with a time constant of 5 μs. Images were acquired with 20 μs pixel dwell time at two different Raman frequencies with different laser powers (vide infra). At first, the tissue was imaged at the Raman frequency of 2700 cm^− 1^ with very low power (< 1.5 mW total at sample plane), and subsequently at 2930 cm^− 1^, which represents -CH_3_ symmetric stretch at 30 mW of total power. The ‘signal’ is defined this way: At 2700 cm^− 1^, there was no signal from the tissue and the only signal obtained was from MWCNT. Note: there is some visible noise in a few air control samples. It is minimal and possibly due to micro debris/dust, as the tissue was uncovered. Nitrogen was streamed continuously over the slides prior to SRS to minimize this issue. The signal from MWCNT was not from the SRS process; and possibly originates from either from two-photon absorption or from thermal response or both. Very low power was used to image just the MWCNT, as at higher power it caused significant photo-damage. Irrespective of the underlying spectroscopic process, the localization of the MWCNT was obtained by overlaying it with the tissue SRS image at 2930 cm^− 1^.

### Lung lavage fluid and plasma cytokine studies

A Meso Scale Discovery multiplex kit (MSD) was used to detect cytokines, which is similar to ELISA, but based on MULTI-ARRAY® technology, a proprietary combination of electrochemiluminescence detection and patterned arrays. The MSD Mouse U-Plex Pro-Inflammatory Panel 1 was used and IFN-γ, IL-10, IL-13, IL-33, IL-1β, TNF-α and IL-6 were measured in the collected lung lavage fluid and plasma.

### RNA analysis

Spleen tissue from control and exposure groups were snap frozen in liquid nitrogen immediately following necropsy. Samples were stored at − 80 °C until analysis. For analysis total RNA was isolated from the frozen spleen tissue using TRIzol Reagent (ThermoFisher Scientific, Grand Island, NY) in accordance with the manufacturer’s protocol. The RNA Integrity Number (RIN) and concentration was confirmed using the RNA 6000 Nano Kit (Agilent Technologies, Santa Clara, CA) and analyzed using an Agilent Bioanalyzer 2000 and spectrophotometric quantification respectively. RNA met the following quality control criteria: RIN > 8.8, A260/280 > 1.8, A260/230 > 1.8. Genomic DNA elimination and first strand cDNA synthesis was performed using RT^2^ First Strand Kit (Qiagen, Valencia, CA) in accordance with the manufacturer’s protocol. Using the Custom RT^2^ Profiler Array (Catalog number PAMM-011Z, Qiagen) samples were assayed for 84 inflammatory cytokines and chemokines to determine the effect of particle exposure on gene expression within the spleen of control and exposed animals. Data analysis, statistical significance and fold change determination was performed using GeneGlobe software suite (Qiagen). All internal quality control standards and conditions were met in accordance with the manufacturer’s analysis protocol.

### Cathepsin assay

Cathepsin activity for the blood plasma was determined by mixing the following assay components in a 96-well plate using PBS as diluent: first blood plasma (50 μl), 2 μg Z-LR-AMC (fluorogenic peptide substrate, R&D systems, Minneapolis, MN) in a total volume of 150 μl. The assays samples were incubated at 37 °C for 1 h, then fluorescence was measured using a plate reader at 380 nm excitation and 460 nm emission.

### Statistical analyses

Statistical analyses involved comparison of means using a one-way *ANOVA* followed by Dunnett’s test or Sidak’s adjustment to compensate for increased type I error resulting from pair-wise mean comparisons. All probabilities were two-tailed unless otherwise stated. Statistical power was greater than 0.8. Statistical significance was defined as a probability of type I error occurring at less than 5% (*P* < 0.05). The minimum number of sample replications was four and the maximum was eight. Graphics and analyses were performed on PRISM v.7.0 (GraphPad, San Diego, CA)

## Supplementary Information

**Additional file 1.**

## Data Availability

The datasets used and/or analyzed during the current study are available from the corresponding author on reasonable request.

## Abbreviations

MWCNT: Multiwalled carbon nanotubes
SRS: Stimulated Raman scattering
H & E: Hematoxylin and eosin
IL: Interleukin
NLRP3: Nod-like receptor protein 3
L-MWCN: Long multiwalled carbon nanotubes
AM: Alveolar macrophage
LLF: Lung lavage fluid
SSC: Side scatter
PMN: Polymorphonuclear neutrophils
APC: Antigen presenting cell
WBC: White blood cell
CD: Cluster of differentiation
CTL: Cytotoxic T lymphocytes
Mono: Monocytes
iMono: Inflammatory monocytes
rMono: Resident monocytes
Mφ: Macrophages
iMφ: Inflammatory macrophages
rMφ: Resident macrophages
Vegfa: Vascular Endothelial Growth Factor A
Cxcr: Chemokine receptor type
Ccr: Chemokine receptor
Ccl: Chemokine ligands
TNF: Tumor necrosis factor
IFN: Interferon
ROS: Reactive oxygen species
TGF: Transforming growth factor
°C: Degrees centigrade
PFA: Paraformaldehyde/phosphate-buffered saline
PBS: Phosphate-buffered saline
SRL: Stimulated Raman loss
μs: Microsecond
RIN: RNA integrity number
SEM: standard error of the mean
