# Supplementary material for: Respiratory and systemic impacts following MWCNT inhalation in B6C3F1/N mice
Source: Part Fibre Toxicol. 2021 Mar 26;18:16. doi: 10.1186/s12989-021-00408-z (PMC7995731; doi:10.1186/s12989-021-00408-z)
Supplement: Supplementary file 1 — Additional file 1. [file 12989_2021_408_MOESM1_ESM.docx]

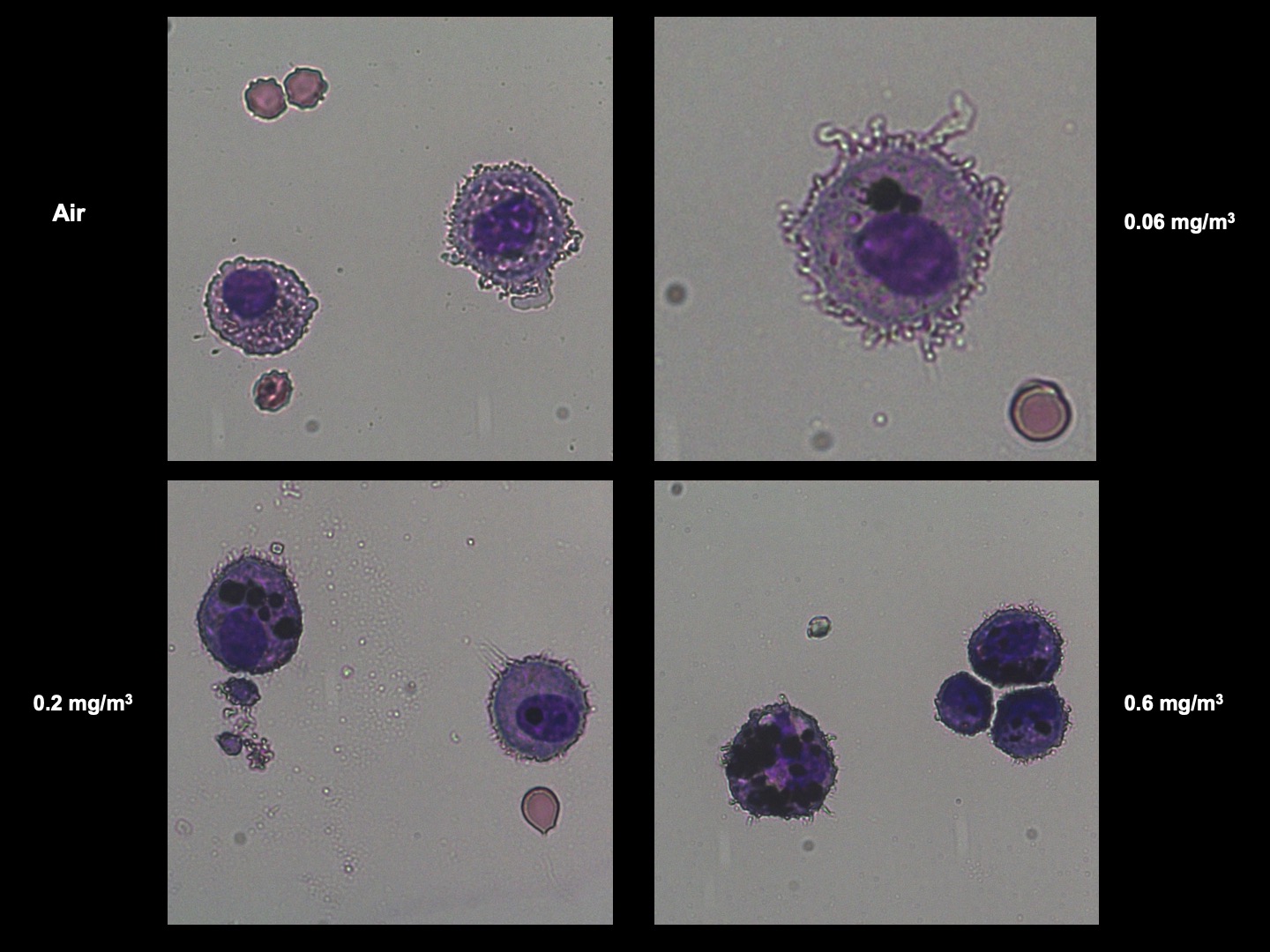


**Supplementary Figure S1:** Isolated alveolar macrophages from the four exposure groups. Cells were centrifuged on to glass slides and stained by Wright-Giemsa. The particle burden was increasing as the dose was increased.


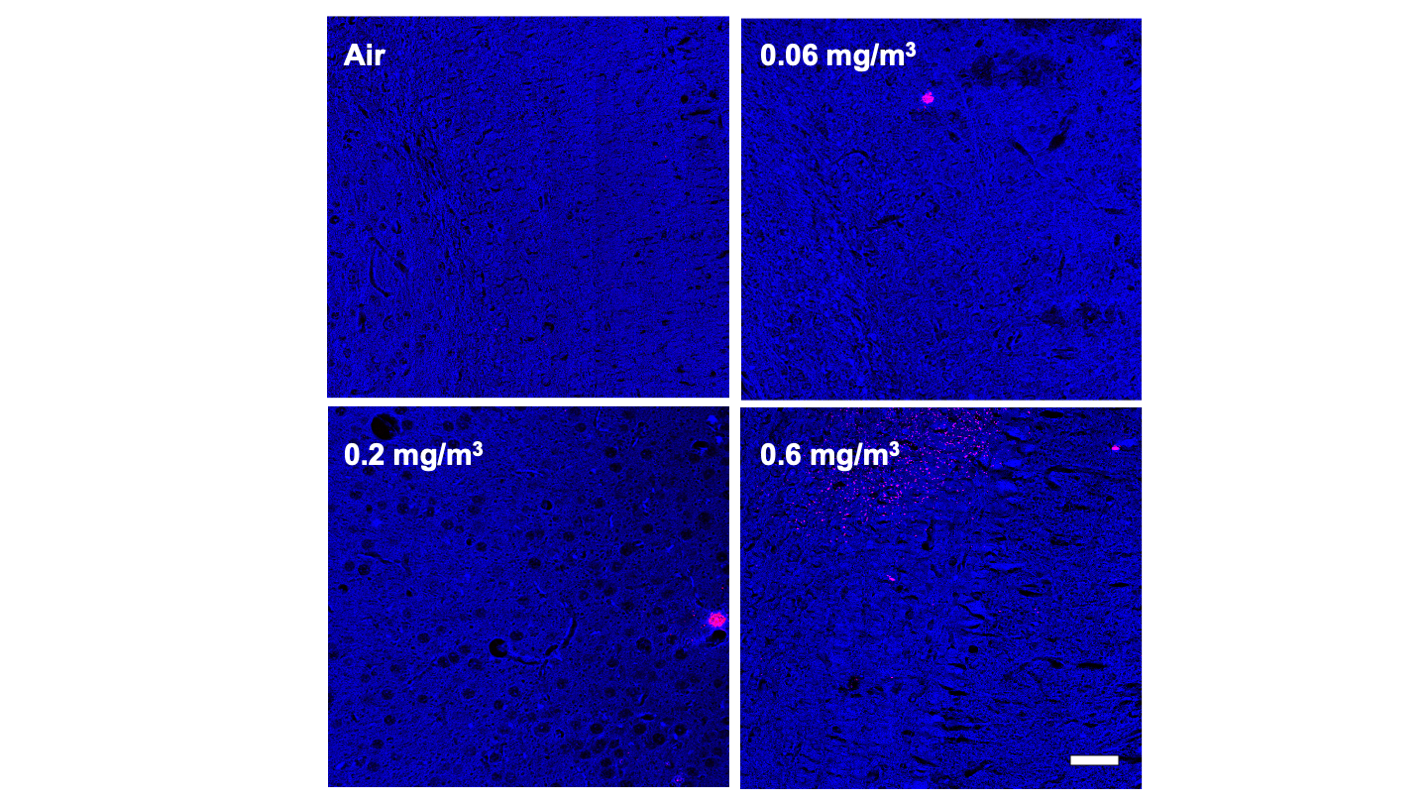


**Supplementary Figure S2:** *Representative MWCNT* *particle deposition in olfactory bulb (OB) tissue following 30-day exposure with all exposure groups shown.* OB tissue was assessed for MWCNT particle deposition using stimulated Raman scattering (SRS) spectroscopy, where the pink color represents detected particles. The above images are representative of sampled OB sections from **A**) Air control or 0 mg/m^3^, **B**) 0.06 mg/m^3^, **C**) 0.2 mg/m^3^, and **D**) 0.6 mg/m^3^. Blue = normal tissue. Pink = MWCNT particle deposition. White scale bar = 50 µm.


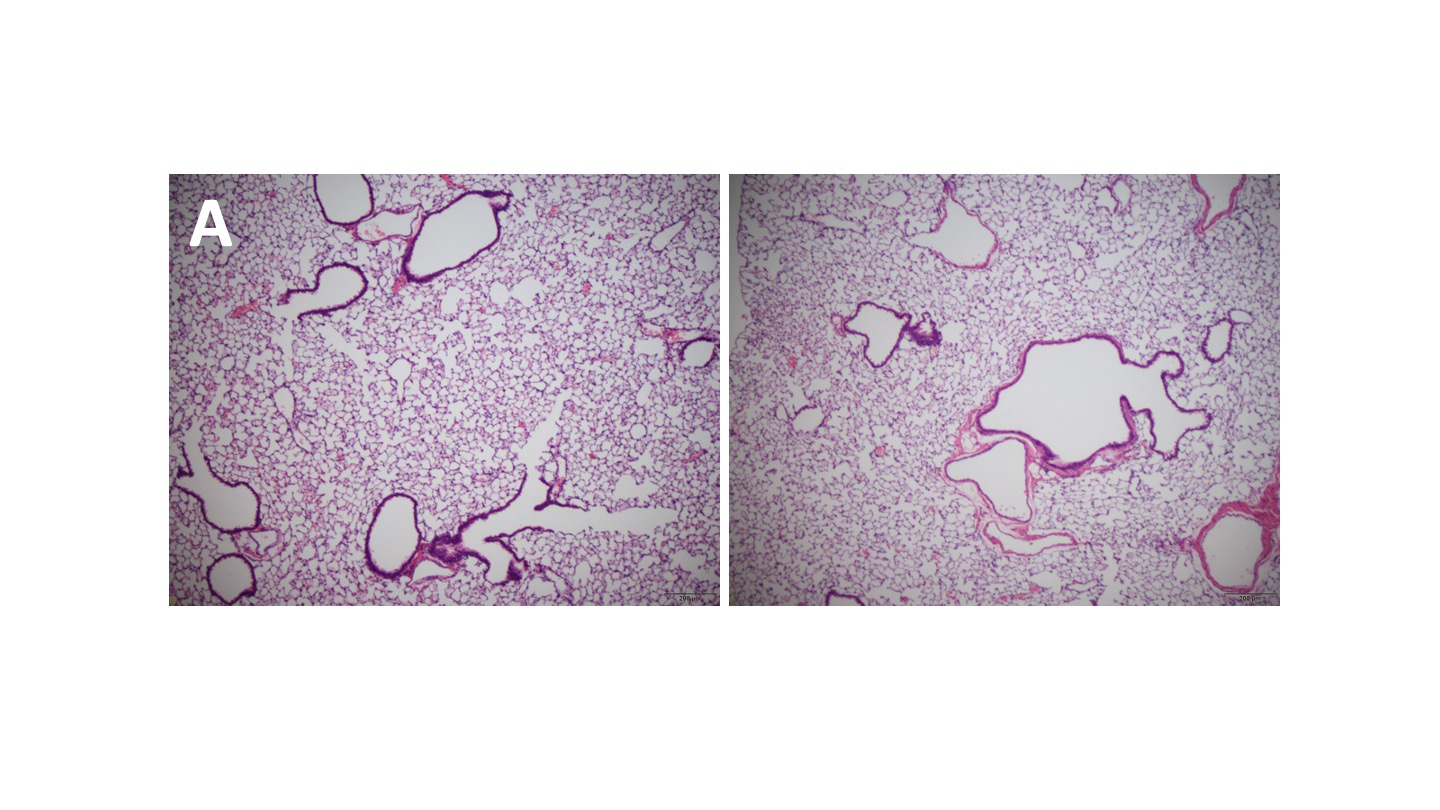


**
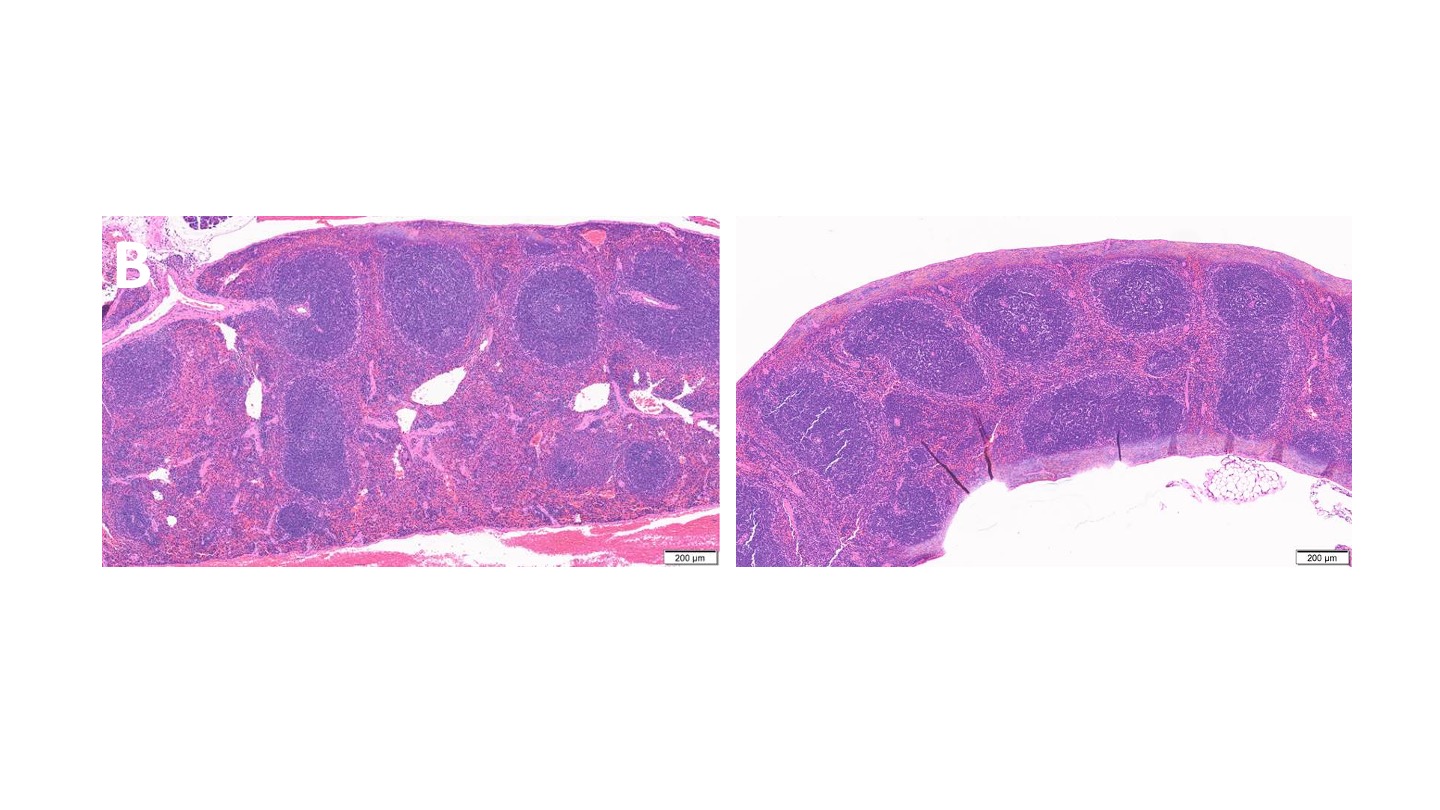
**

**
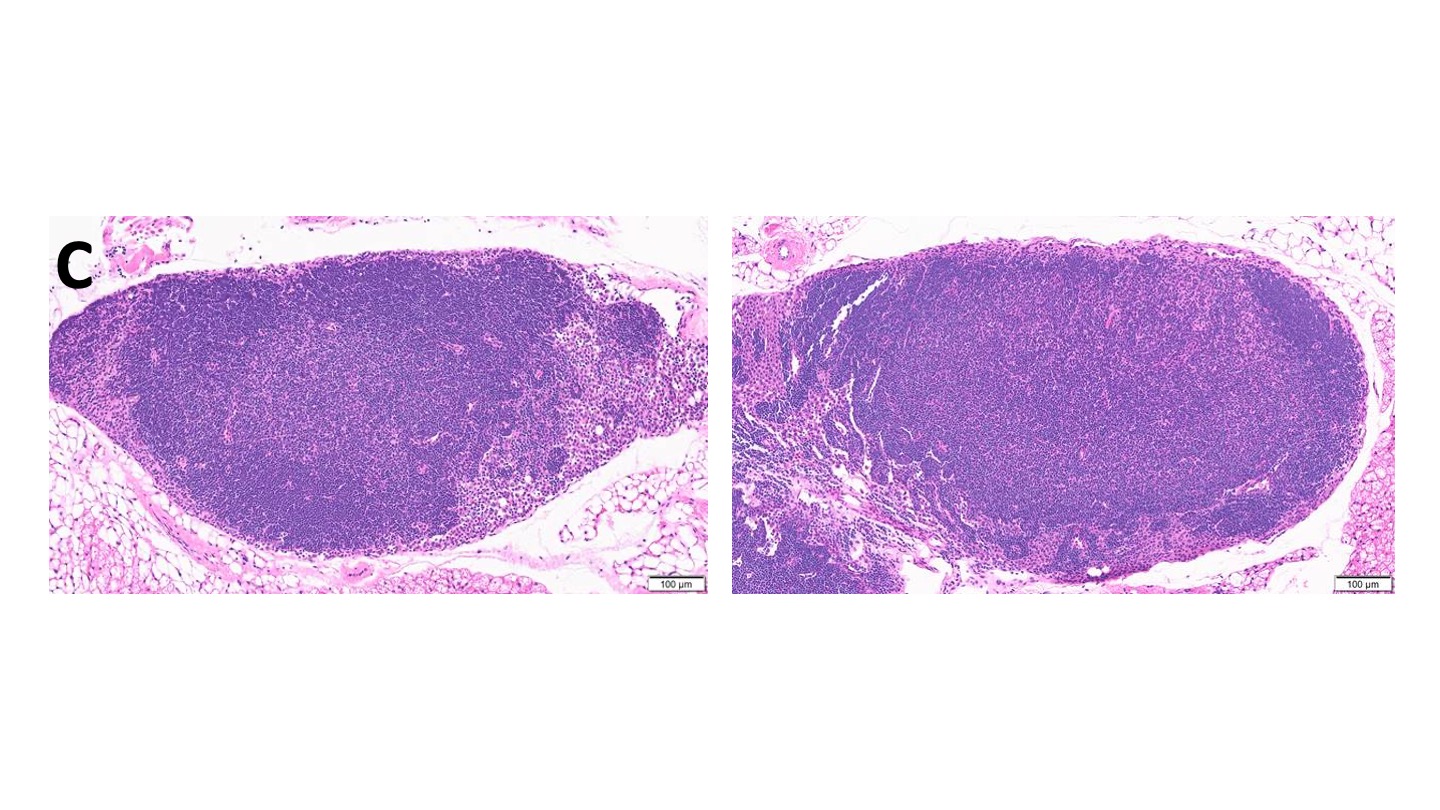
**

**
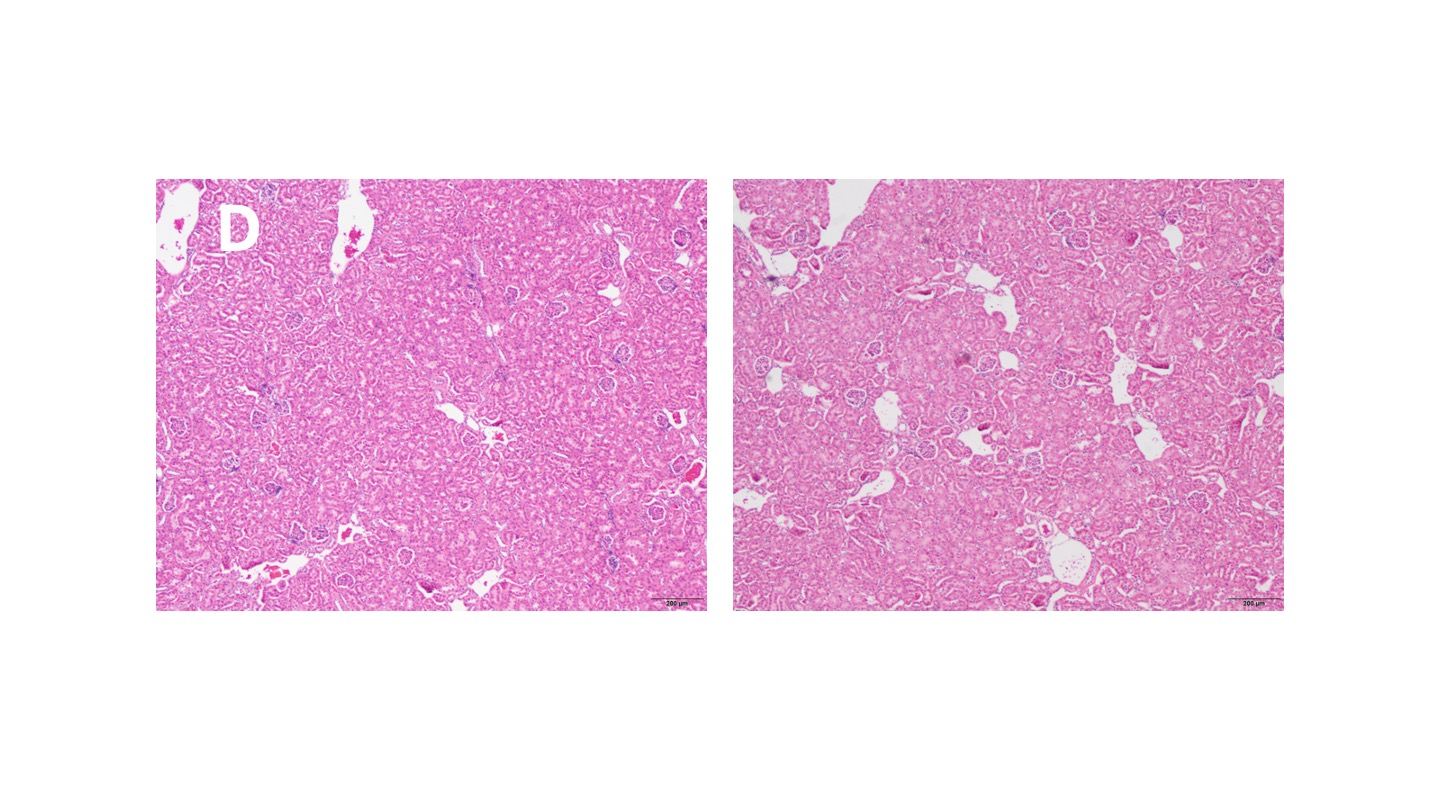
**

**
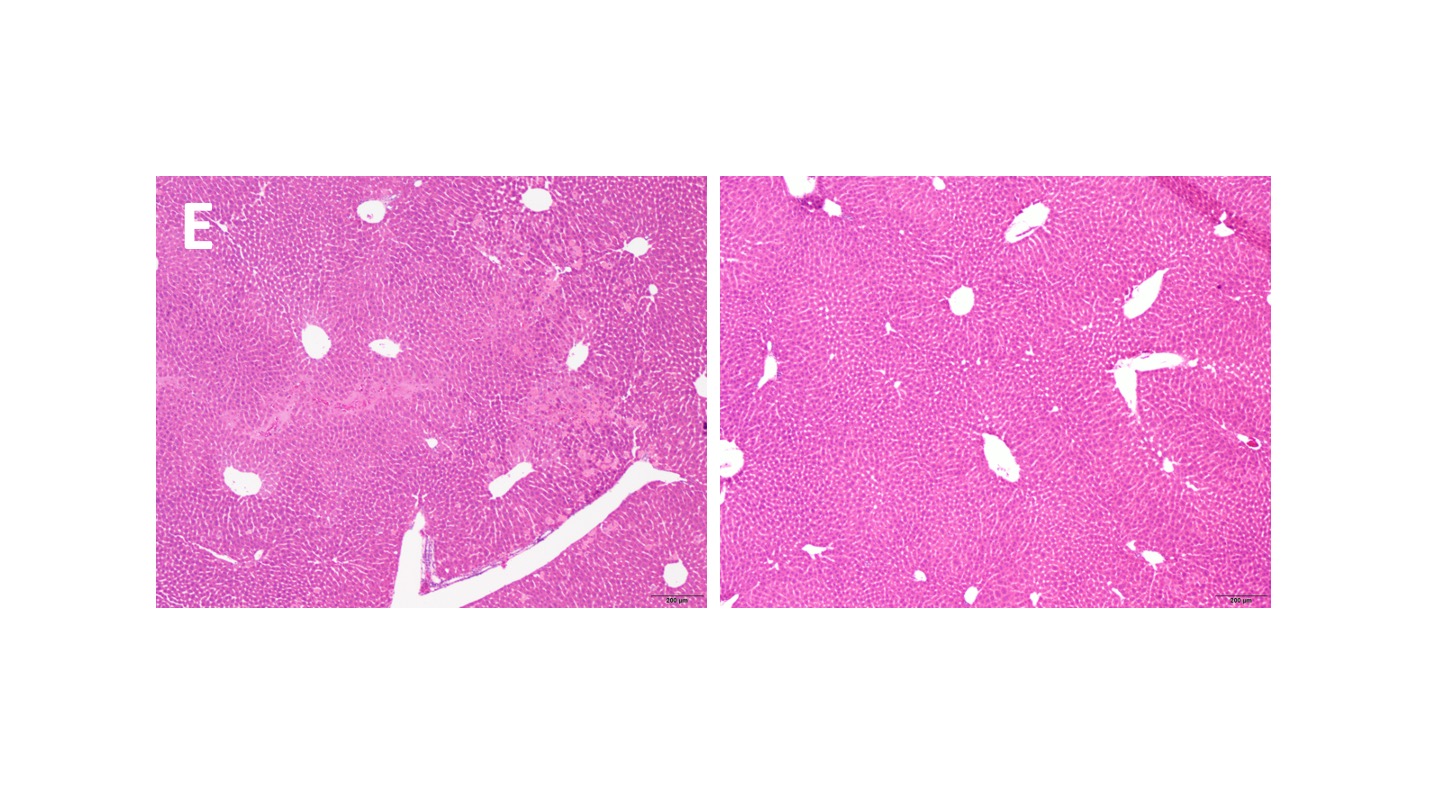
**

**
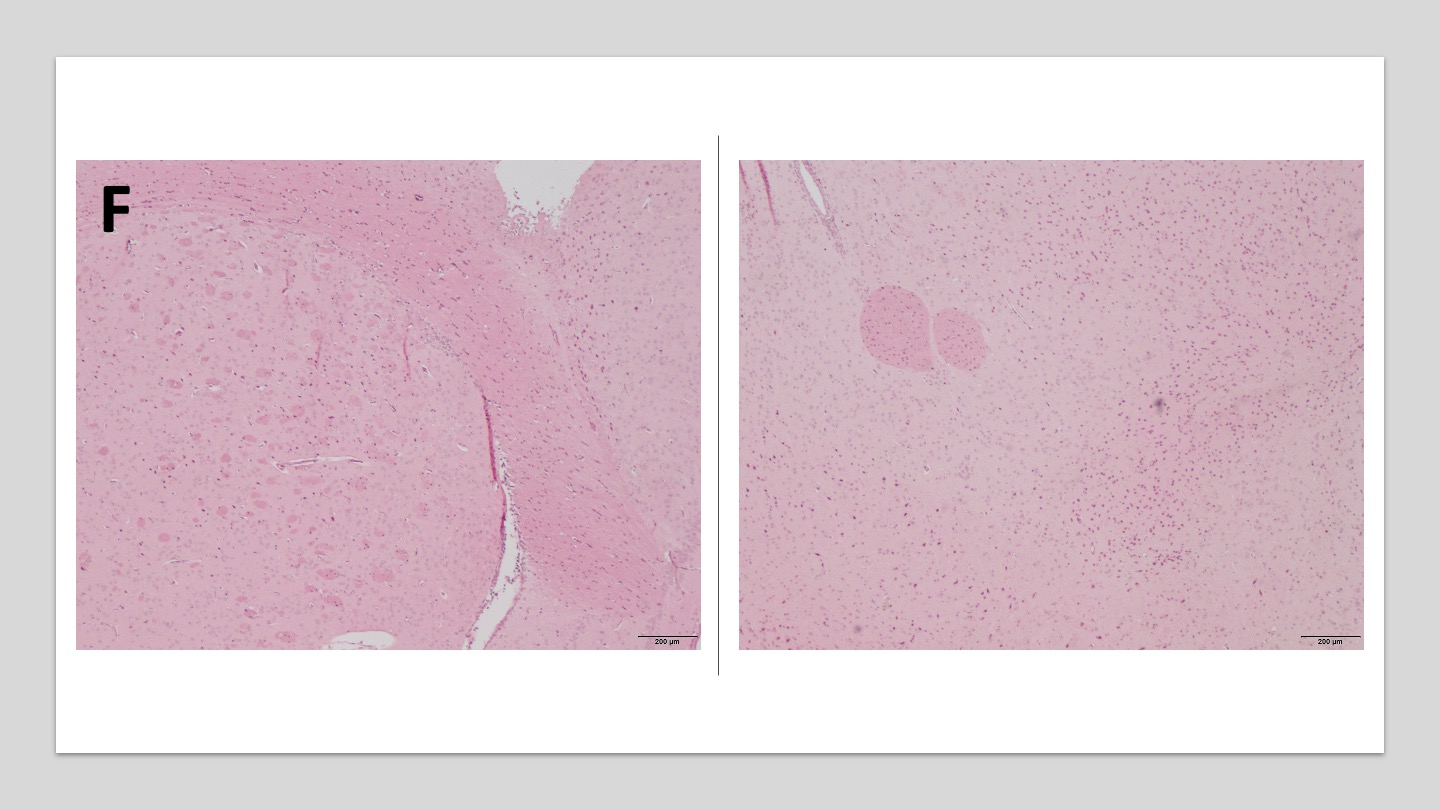
**

**Supplementary Figure S3:** Low magnification H & E-stained tissue from A) Lung, B) Spleen, C) Brachial Lymph Nodes, D) Kidney, E) Liver, and F) Olfactory Bulb from air control mouse (left panel) and high dose MWCNT (right panel) respectively.

**Table S1:** Myeloid population surface markers

| **Marker** | **CD45** | **CD11b** | **CD11c** | **F4/80** | **MHCII** | **Ly6G** | **Ly6C** | **Siglec F** | **SSC** |
| --- | --- | --- | --- | --- | --- | --- | --- | --- | --- |
| **Neutrophils** | + | + | - | - | - | + | + | +/- | int |
| **Macrophages (M0)** | + | + | - | +/- | + | - | +/- | - | int |
| **Resident M0** | + | + | - | +/- | + | - | lo | - | int |
| **Inflammatory M0** | + | + | - | +/- | + | - | hi | - | int |
| **Monocytes** | + | + | - | +/- | - | - | +/- | - | int |
| **Resident Monos** | + | + | - | +/- | - | - | lo | - | int |
| **Inflammatory Monos** | + | + | - | +/- | - | - | hi | - | int |
| **CD11b+ DCs** | + | + | + | + | + | - | lo | - | int |
| **CD11b- DCs** | + | - | + | +/- | + | - | lo | - | int |
| **Eosinophils** | + | + | - | - | - |  | + | + | hi |

**Table S2:** Lymphoid population surface markers

| **Marker** | **CD45** | **CD3** | **CD19** | **CD4** | **CD8** | **CD44** |
| --- | --- | --- | --- | --- | --- | --- |
| **B cells** | + | - | + | - | - | N/A |
| **T cells** | + | + | - | +/- | +/- | +/++ |
| **Cytotoxic Lymphs (CTL)** | + | + | - | - | + | +/++ |
| **T helper cells** | + | + | - | + | - | +/++ |
| **Activated T helper cells** | + | + | - | + | - | ++ |
| **Activated CTL** | + | + | - | - | + | ++ |

**Table S3:** Flow Cytometry reagents/Titrations

| **Lymphoid**  **Panel** | **Specificity** | **CD45** | **CD4** | **CD8a** | **CD44** | **CD19** | **CD40** | **CD3** |  |
| --- | --- | --- | --- | --- | --- | --- | --- | --- | --- |
|  | Clone | 30-F11 | RM4-5 | 53-6.7 | IM7 | 6D5 | 3/23 | 17A2 |  |
|  | Format | BV510 | FITC | PerCPCy5.5 | PE | PEDzl594 | PE-Cy7 | APC |  |
|  | Vendor | BioLegend | Tonbo | Tonbo | Tonbo | BioLegend | BioLegend | Tonbo |  |
|  | Cat # | 103138 | 35-0042 | 65-0081 | 50-0441 | 115554 | 1246022 | 20-0032 |  |
|  | Titration | 1:100 | 1:1600 | 1:400 | 1:1000 | 1:500 | 1:400 | 1:100 |  |
|  |  |  |  |  |  |  |  |  |  |
| **Myeloid**  **Panel** | **Specificity** | **CD45** | **MHCII** | **CD11b** | **F4/80** | **Ly6C** | **CD11c** | **Ly6G** | **Siglec F** |
|  | Clone | 30-F11 | M5/114.15.2 | M1/70 | BM8.1 | HK1.4 | N418 | 1A8 | E50-2440 |
|  | Format | BV510 | FITC | PerCPCy5.5 | PE | PEDzl594 | APC | AF700 | APC-Cy7 |
|  | Vendor | BioLegend | Tonbo | Tonbo | Tonbo | BioLegend | Tonbo | BioLegend | BD BioSci |
|  | Cat # | 103138 | 35-5321 | 65-0112 | 50-4801 | 128044 | 20-0114 | 127622 | 565527 |
|  | Titration | 1:100 | 1:800 | 1:400 | 1:200 | 1:100 | 1:100 | 1:100 | 1:100 |
|  |  |  |  |  |  |  |  |  |  |
| **Other** | **Specificity** | **CD16/32** | **Live/Dead** | **AbC Comp** |  |  |  |  |  |
|  | Clone | 2.4G2 | N/A | N/A |  |  |  |  |  |
|  | Format | N/A | DAPI | N/A |  |  |  |  |  |
|  | Vendor | Tonbo | Invitrogen | ThermoFisher |  |  |  |  |  |
|  | Cat # | 70-0161 | D3571 | A10497 |  |  |  |  |  |
|  | Titration | 1:100 | 1:10,000 | NA |  |  |  |  |  |

Attune NxT Acoustic Focusing Flow Cytometer – Life Technologies

Flow cell: Quartz cuvette gel coupled to 1.2 numerical aperture (NA) collection lens, 200 x 200 μm

Laser profile: 10 x 50 μm flat-top laser with fixed alignment

Configuration

(only showing lasers/detectors used)

| **405 nm - 50mW*** | | **488 nm – 50 mW*** | | **561 nm – 50 mW*** | | | **633 nm – 100 mW*** | | |
| --- | --- | --- | --- | --- | --- | --- | --- | --- | --- |
| 417 LP | 495 LP | 503 LP | 650 LP | 577 LP | 600 LP | 740 LP | 654 LP | 690 LP | 740 LP |
| 440/50 BP | 512/25 BP | 530/30 BP | 695/40 BP | 585/16 BP | 620/15 BP | 780/60 BP | 670/14 BP | 720/30 BP | 780/60 BP |
| DAPI | BV510 | FITC | PerCPCy5.5 | PE | PEDzl594 | PE-Cy7 | APC | AF700 | APC-Cy7 |

*Amount of measured usable laser power after light has gone through the beam optics and shaping filters

**Table S4:** Genes altered in the spleen by MWCNT inhalation

| Gene | Fold Change | Related protein – related functions | Significance at *P* < 0.05 | Direction |
| --- | --- | --- | --- | --- |
| Vegfa | 1.43 | Growth factor - proliferation and migration of vascular endothelial cells physiological and pathological angiogenesis | *0.028 | Up |
| Aimp1 | 0.16 | Cytokine - induced by apoptosis, angiogenesis, inflammation, and wound healing | 0.6 | Down |
| Ccl19 | 6.42 | Cytokine - trafficking of T cell and B cell migration to secondary lymphoid organs | 0.51 | Up |
| Ccl2 | 0.28 | Chemokine - monocytes and memory T cells but not for neutrophils | *0.01 | Down |
| Ccl7 | 0.29 | Chemotactic protein 3 - attracts macrophages during inflammation and metastasis | *0.018 | Down |
| Ccl9 | 2.01 | Part of the CC chemokine family. It is also called MIP-1γ, MRP-2 and CCF18, macrophage attractant | 0.176 | Up |
| Ccr2 | 2.68 | Monocyte chemoattractant protein - mediates monocyte chemotaxis and monocyte infiltration in inflammatory diseases | 0.062 | Up |
| Ccr8 | 0.35 | Receptor - may contribute to the proper positioning of activated T cells within the antigenic challenge sites and specialized areas of lymphoid tissues | *0.041 | Down |
| Cxcl1 | 0.37 | Chemokine - chemoattractant for neutrophils. | 0.215 | Down |
| Cxcl5 | 0.44 | Protein - binds the G-protein coupled receptor chemokine (C-X-C motif) receptor 2 to recruit neutrophils, promotes angiogenesis and remodels connective tissues | 0.144 | Down |
| Cxcr2 | 9.16 | Receptor - mediates neutrophil migration to sites of inflammation | 0.563 | Up |
| Vegfa | 1.46 | See above | *0.028 | Up |
| Ccl19 | 19.62 | See above | 0.333 | Up |
| Ccr2 | 3.25 | See above | *0.05 | Up |
| Ccr3 | 2.83 | Receptor - activation of eosinophils and other inflammatory cells in the allergic airway | *0.042 | Up |
| Ccr5 | 2.73 | Receptor – ligands include monocyte chemoattractant protein 2 (MCP-2), macrophage inflammatory protein 1 alpha (MIP-1 alpha), macrophage inflammatory protein 1 beta (MIP-1 beta) | 0.118 | Up |
| Cxcr2 | 11.06 | See above | 0.38 | Up |
| Vegfa | 2.49 | See above | *0.045 | Up |

Blue = 0.06 mg/m^3^ dose compared to Air control

Red = 0.2 mg/m^3^ dose compared to Air control

Green = 0.6 mg/m^3^ dose compared to Air control
